# Mapping the range of policies relevant to care of small and nutritionally at-risk infants under 6 months and their mothers in Ethiopia: a scoping review protocol

**DOI:** 10.1136/bmjopen-2022-069359

**Published:** 2023-09-19

**Authors:** Marie McGrath, Mirkuzie Woldie, Melkamu Berhane, Mubarek Abera, Endashaw Hailu, Ritu Rana, Betty Lanyero, Carlos Grijalva-Eternod, Alemseged Abdissa, Tsinuel Girma, Marko Kerac, Tracey Smythe

**Affiliations:** 1 Emergency Nutrition Network, Kidlington, UK; 2 Faculty of Epidemiology and Population Health, London School of Hygiene and Tropical Medicine, London, UK; 3 Maternal and Child Health and Nutrition Directorate, Ethiopia Ministry of Health, Addis Ababa, Ethiopia; 4 Department of Pediatrics, Jimma University, Jimma, Ethiopia; 5 Faculty of Medical Sciences, Jimma University, Jimma, Ethiopia; 6 GOAL Ethiopia, Addis Ababa, Ethiopia; 7 Department of Public Health Programmes, Indian Institute of Public Health Gandhinagar, Gandhinagar, Gujarat, India; 8 Emergency Preparedness and Response Unit, World Health Organization Country Office for Ethiopia, Addis Ababa, Ethiopia; 9 Institute for Global Health, University College London, London, UK; 10 Armauer Hansen Research Institute, Addis Ababa, Ethiopia; 11 Department of Pediatrics and Child Health, Jimma University, Jimma, Ethiopia; 12 Faculty of Epidemiology and Population Health, International Centre for Evidence in Disability, Department of Population Health, London School of Hygiene and Tropical Medicine, London, UK

**Keywords:** nutrition, health policy, international health services, public health

## Abstract

**Introduction:**

Evidence gaps limit management of small and/or nutritionally at-risk infants under 6 months and their mothers, who are at higher risk of death, illness, malnutrition and poor growth and development. These infants may be low birth weight, wasted, stunted and/or underweight. An integrated care model to guide their management (MAMI Care Pathway) is being tested in a randomised controlled trial in Ethiopia. Evaluating the extent to which an innovation is consistent with national policies and priorities will aid evidence uptake and plan for scale.

**Methods and analysis:**

This review will evaluate the extent to which the MAMI Care Pathway is consistent with national policies that relate to the care of at-risk infants under 6 months and their mothers in Ethiopia. The objectives are to describe the range and characteristics, concepts, strategic interventions, coherence and alignment of existing policies and identify opportunities and gaps. It will be conducted in accordance with the JBI methodology for scoping reviews (PRISMA-ScR). Eligible documents include infant and maternal health, nutrition, child development, food and social welfare-related policies publicly available in English and Amharic. The protocol was registered on the Open Science Framework Registry on 20 June 2022 (https://osf.io/m4jt6).

Grey literature will be identified through government and agency websites, national and subnational contacts and Google Scholar, and published policies through electronic database searches (MEDLINE, EMBASE and Global and Health Information). The searches will take place between October 2023 and March 2024. A standardised data extraction tool will be used. Descriptive analysis of data will be undertaken. Data will be mapped visually and tabulated. Results will be described in narrative form. National stakeholder discussions will inform conclusions and recommendations.

**Ethics and dissemination:**

Ethical approval is not required as data consist solely of publicly available material. Findings will be used to evidence national and international policy and practice.

**STRENGTHS AND LIMITATIONS OF THIS STUDY**

- A scoping review is most suited to identifying relevant policies given they are characteristically diverse and most likely sourced from in-country grey literature, including through contacts and networks, rather than electronic databases.
- National stakeholders who are directly engaged in policy development and research in Ethiopia are directly involved in the review from conceptualisation onwards.
- The review is consistent with the stated strategic intent of the Ethiopian government for multisectoral integration of nutrition.
- Critical appraisal of the identified policies is necessary to practically inform national stakeholders and to evidence uptake activities related to planned research in Ethiopia.
- The review is limited to English and Amharic documentation.

## Introduction

Globally, about one in five infants under 6 months is small and/or nutritionally at-risk (at-risk).^1^ An estimated 20.1% (23.8 million) infants under 6 months are underweight (low weight-for-age), 17.6% (24.5 million) are wasted/acutely malnourished (low weight-for-length) and 17.6% (21.5 million) are stunted (low length-for-age).^1^ An estimated 9.76% of babies are born low birth weight (LBW) in Sub-Saharan Africa.^2^ In Ethiopia, an estimated 7% of children under 5 years are wasted and 36.8% are stunted,^3^ while an estimated 13.2% are born LBW.^4^ These infants are at higher risk of death, illness, malnutrition and poor growth and development.^5 6^ Furthermore, experiencing an episode of wasting in early life leads to an increased risk of further wasting in later life^6^ which contributes to the global burden of 47 million wasted children.^7^

Community-based management of wasting in children over 6 months of age is a well-established approach globally, involving outpatient and decentralised care for medically uncomplicated cases.^8^ Equivalent services for infants under 6 months are not common in low-income and middle-income countries for many reasons, including assumptions that infants under 6 months are protected from malnutrition through exclusive breastfeeding.^9^ In reality, exclusive breastfeeding prevalence is suboptimal in almost all countries^10^ and may be overestimated.^11^ In Ethiopia, prevalence among infants under 6 months is estimated at 60.4%, with regional variations.^12^

Increased awareness of the significant burden of care in this age group led to updated WHO guidelines in 2013 recommending outpatient care for uncomplicated severely wasted/acutely malnourished infants under 6 months.^13^ However, uptake of WHO recommendations into national guidance has been low and slow. A 2020 review of 63 national protocols for malnutrition treatment found that only six countries recommended outpatient care for infants under 6 months.^14^ In most countries, including Ethiopia, inpatient treatment remains the only option, which is less accessible for families, limits service coverage and may not fully cater for their needs.^15^ Barriers to national policy uptake of WHO guidelines include demand for more context-specific evidence of what works, for whom and at what cost for health systems, services, communities, families and mothers.^16^ This means that most at-risk infants and their mothers do not receive the prompt care they need, particularly after the early postnatal period (ie, post 6 weeks to 6 months of age), missing a critical window for early treatment and preventive action.

Longstanding gaps in implementation guidance on how best to identify and manage at-risk infants under 6 months in outpatient and community services have hampered WHO policy translation into practice.^9^ To help address this, the MAMI Care Pathway Package^17^ was developed through expert consultation by the MAMI Global Network, an established global collaboration of nutrition and health practitioners, researchers and experts (www.ennonline.net/ourwork/research/mami). This resource material applies a care pathway model to guide practical management of at-risk infants under 6 months and their mothers in pursuit of quality, respectful care continuity across systems of health and nutrition. Support to the mother–infant pair is central to the MAMI approach. It bridges maternal and child nutrition, health and social interventions to support continuity of quality, respectful care, integrated into and building on existing health and nutrition systems and services wherever possible. For example, it includes basic screening for at-risk infants under 6 months and mothers at routine immunisation visits and supports implementation of WHO Integrated Management of Childhood Illness guidelines in accordance with local protocols. The MAMI Care Pathway Package has been implemented in multiple contexts by Non-Governmental Organisations (NGOs) through small-scale programmes and operational research.^18–20^ There have been two updates (most recently in 2021) through expert consultation and informed by implementation experiences and the latest evidence.

Global ambitions for integrated services across sectors are shared by the Ethiopian government. The Ethiopian National Food and Nutrition Strategy, for example, highlights multisectoral coordination and integration as critical, but notes that progress has been slow and ineffective in bringing about changes to nutrition and public health problems. The Strategy suggests this is partly due to inadequate mainstreaming of nutrition into relevant sectoral policies, strategies, programmes and operational plans.^21^ Sectoral policy development processes and roll-out plans are often siloed in different departments, ministries and institutions, thus missing opportunities to align and collaborate. The concept of at-risk infants under 6 months and their mothers may be defined and understood in different ways across diverse policymakers, which may itself impede cross-sectoral collaboration. Poor alignment of policy limits achievement of strategic ambition and misses opportunities to leverage collection action. Lack of policy coherence also influences the operational context in which health workers work; siloed services may restrict frontline workers’ autonomy and capacity to innovate and connect across services during their day-to-day activities.

To help fill the critical evidence gap in outpatient care, the MAMI Care Pathway is being tested in Ethiopia in a north–south research partnership that includes a pragmatic randomised controlled trial, formative research and a realist evaluation (MAMI RISE) (www.ennonline.net/ourwork/research/mamiriseethiopia). Findings will be used to evidence national and international policy and practice, with the goal of achieving scalable sustainable care for at-risk infants under 6 months and their mothers. Understanding, accounting for and responding to the national policy context are critical to ensure that the evidence generated meets the needs of national decision-makers^22 23^ and to support the research uptake process. Interventions that correspond to national health sector goals are likely to gain the political and administrative support necessary for larger-scale implementation if the project results demonstrate success.^24^ Evaluating the extent to which the innovation is consistent with existing policies, regulations, national health plans and priorities is a key step in early planning for future scale.^24^ To this end, a scoping review will be undertaken to describe and appraise policies in Ethiopia that address the multiple dimensions of care, including nutrition, health, child development, food and social welfare, through the lens of managing at-risk infants under 6 months and their mothers.

## Methods and analysis

### Design

A scoping review methodology has been selected to allow the collation of a diverse range of relevant information not previously merged in this way.^25^ The proposed scoping review will be conducted in accordance with the JBI methodology for scoping reviews.^26^ We have used the JBI Population, Concept and Context strategy to define the title, scoping review objective, scoping review question and inclusion criteria. This review will focus primarily on policy content that will contribute to broader policy analysis that considers policy context, process and actors^27^ to support translation of evidence to policy in the context of Ethiopia.^28^

### Aim and objectives

The aim of this scoping review is to evaluate the extent to which the MAMI Care Pathway is consistent with national policies that relate to the care of at-risk infants under 6 months and their mothers in Ethiopia. The objectives of the review are to:

1. Describe the range and characteristics of existing policy documents relevant to the care of small and/or nutritionally at-risk infants under 6 months and their mothers.
2. Describe how small and/or nutritionally at-risk infants under 6 months and their mothers are conceptualised/defined in policies.
3. Identify strategic interventions targeted at the care of small and/or nutritionally at-risk infants under 6 months and/or mothers; and
4. Assess alignment, coherence, opportunities and gaps within and across the identified policies.

Mapping the range and characteristics of national policies through the lens of at-risk infants under 6 months and their mothers in Ethiopia will help identify barriers to address, opportunities to leverage and gaps to fill to help mainstream integrated continuity of care across multisectoral national policy and service provisions. This review will inform research dissemination actions and aid evidence uptake. Furthermore, it will support the delivery and development of the strategic priorities of the Ethiopian government, including implementation of the Ethiopian National Food and Nutrition Strategy. It will also inform early planning by national decision-makers and help their appraisal of potential for scaling sustainable integrated care of at-risk infants under 6 months^24^ in the future.

### Review questions

1. What is the range and characteristics of policies in Ethiopia that relate to the care of small and/or nutritionally at-risk infants under 6 months and their mothers?
2. How are small and/or nutritionally at-risk infants under 6 months and their mothers conceptualised, described, or defined in Ethiopian policies?
3. What strategic interventions targeted at the care of small and/or nutritionally at-risk infants under 6 months and mothers are outlined in the identified policies?
4. What alignment, coherence, opportunities and gaps are there in identified policies for the care of small and/or nutritionally at-risk infants under 6 months and their mothers in Ethiopia?

All relevant infant and maternal health, nutrition, child development, food and social welfare-related policies and policy documents will be included. To be considered as a policy or policy-related document, we will consider the description provided by Haynes *et al*^25^ (box 1). The searches will take place between October 2023 and March 2024.

Box 1 **Policy definitions**

Policy: A formal statement or action plan developed by a government agency or statutory body in response to an identified problem. This includes state-wide or national legislation, policies, programmes, directives, protocols, guidelines and service models.

Policy: A formal statement or action plan developed by a government agency or statutory body in response to an identified problem. This includes state-wide or national legislation, policies, programmes, directives, protocols, guidelines and service models.

Policy document: A review, report, discussion paper, draft or final policy, formal directive, programme plan, strategic plan, ministerial brief, budget twice daily, service agreement, implementation plan, guideline or protocol with a focus on health service or programme design, delivery, evaluation, or resourcing.

### Population (participants)

This scoping review will focus on individuals (mothers, infants under 6 months, including neonates) across subpopulations (ethnicities, communities) and society in Ethiopia.

Our interest is in those infants under 6 months who are born small (eg, LBW, premature, small-for-gestational age) and/or are identified as malnourished or growing poorly or at-risk of malnutrition or poor growth, rather than the general population of all infants. We include those with: LBW, prematurity, small-for-gestational age, growth faltering, low weight-for-length, low weight-for-age, low length-for-age, low mid upper arm circumference and those whose mothers have physical/mental health issues that may affect the infant’s care or feeding. These criteria reflect a working definition applied in the MAMI Care Pathway Package.^17^ We will also be open to other definitions of at-risk infants under 6 months—the review will help us understand different definitions and links between them better.

### Concept

This scoping review of policies aims to identify the main documents that have a bearing on the nature of practice regarding the care of at-risk infants under 6 months and their mothers in Ethiopia. This review aims to capture all current infant and maternal nutrition, health, child development, food and social welfare-related policy evidence in Ethiopia. We wish to identify policy provisions for both direct and broader/indirect support for the care of at-risk mother–infant pairs. Policies that address treatment and/or prevention and that address maternal well-being, economic security and social enterprise, such as social protection and food assistance, will be included in the review. The policy characterisation will conceptually map definitions used, that is, describe the concepts and terminology used to define the population being examined. Policies will be appraised to identify alignment, coherence, opportunities and gaps. Opportunities investigated will include information on policy development processes, while the degree to which details (scope and purpose, stakeholder involvement and procedures for update) are available will be reported.

### Context

All settings and contexts will be considered eligible that apply to the care of at-risk infants under 6 months and their mothers in Ethiopia. These include but will not be limited to inpatient care, outpatient care, primary healthcare, communities and households/families.

### Inclusion criteria

Health, nutrition, food, child development, social welfare and other relevant policies in Ethiopia that make provision for or that are applicable to the care of at-risk infants under 6 months and/or their mothers will be included. National policy documents and WHO, United Nations, NGO and funder policies will also be included. Documents published in English and Amharic will be included. No time limit will be set.

### Exclusion criteria

Documents focusing on countries others than Ethiopia will be excluded from the review. Documents focusing on the general population of infants under 6 months who are not at-risk and that are not applicable to at-risk infant/mothers’ care will also be excluded.

### Search strategy

The development of the search strategy is taking place in collaboration with a librarian. A pilot test of source selectors will be conducted whereby a random sample of abstracts will be screened by the selection team using the eligibility criteria. Discrepancies will be discussed and clarifications/modifications made to eligibility criteria/definitions accordingly. A preliminary search of Cochrane Library, MEDLINE (Ovid), PROSPERO and Open Science Framework (OSF) Registry for systematic and scoping reviews of policies in Ethiopia was conducted on 26 May 2022 and no reviews were found on this review topic. The protocol was registered on the OSF Registry on 20 June 2022 (https://osf.io/m4jt6).

The search strategy will be carried out using the search terms related to each of the concepts of interest. The following search terms and their variants will be used, as split across six concepts: small, nutritionally at-risk, infants under 6 months, mothers, policies and Ethiopia. The search strategy will combine keywords to include ‘small’ OR ‘nutritionally at-risk’ AND ‘infant under six months’ OR ‘mothers’ AND ‘policies' in 'Ethiopia’. A series of searches will be performed towards a final search to identify policies that relate to small and/or nutritionally at-risk infants under 6 months and their mothers in Ethiopia. See Online supplemental file 1 for the initial search terms (Medical Subject Headings (MeSH) terms and key words) using MEDLINE (Ovid) platform that will be further adapted to other databases/grey literature sources as described below.

10.1136/bmjopen-2022-069359.supp1 Supplementary data

The scoping review will involve the identification of publicly available grey literature and a comprehensive search of three electronic databases for published policies. We anticipate that grey literature will be the primary source of content.

Findings will be reported according to the Preferred Reporting for Items for Systematic reviews and Meta-Analysis Protocols (PRISMA-P) and extension for scoping review (PRISMA-ScR). The PRISMA-ScR checklist will be included in the review results paper. Patients and the public were not involved in the preparation of this protocol.

### Grey literature search

Search terms will be applied as appropriate (see online supplemental file 1) and additional search terms iteratively developed and used to capture policies that do not specify the concepts of interest and reported on. Google Scholar will be used to identify the grey literature and relevant policy documents, reviews or executive summaries. Websites and contacts of the Ethiopian government, MAMI RISE Research Team, WHO, UNICEF, World Food Programme, in-country agencies and funders will be used to acquire relevant information and documents. Policies sourced by the MAMI RISE Research Team during the formative phase of the research programme, including those identified in stakeholder interviews as part of a feasibility study of the MAMI Care Pathway, will be included/updated with the latest version as appropriate. Grey literature/database searches will be conducted by one researcher and sources and findings cross-checked by a second national researcher. The search process will be mapped and reported. The reference lists of all included literature will be additionally hand-searched for any further relevant literature.

### Electronic database search

Publications related to policy provision for maternal and infants under 6 months care across sectors and services in Ethiopia will be sought using online search engines: MEDLINE (Ovid), EMBASE and Global and Health Information. Search terms will be applied and adapted as appropriate to the syntax of each database. Key research terms will also comprise those obtained via the subject headings of searched databases (eg, MeSH; see online supplemental file 1 for MEDLINE (Ovid) search terms). The search strategies for each database will be similar in structure, with similar search terms and synonyms. Additional keywords and sources identified during the process will be noted and reported.

### Data extraction

All citations of literature to be included in the review will be uploaded into EndNote for deduplication. Results will be exported to Rayan for screening by two independent reviewers. Following an initial pilot, each reviewer will independently and systematically screen the title and abstracts of all documents using the inclusion criteria to do so. Documents for review will be limited to the first 50 texts. The full text of documents for possible inclusion in the review will be assessed by the screening team at a reviewers’ meeting. Reasons for exclusion of sources of evidence at full-text review will be logged and then stated in the review itself.

A standardised data extraction tool will be used against the selected policies (see the draft charting table in Table 1). Extracted data will be organised applying predetermined fields and the themes arising. Basic coding of data to categories, such as sectors, subpopulation and by policy type and source, will be conducted. Concepts/definitions within a field and policy development information will be extracted. One researcher will carry out data extraction and the data will be assessed by a second researcher. The data extraction tool will be changed and adapted during the process of gathering information from the respective evidence source, and all modifications made will be explained fully in the final review.

**Table 1:** Draft charting table for data extraction

Parameters	Results (n, %)
Policy characteristics	
No. of policies identified	Total number
Publication date	<2 years (to current date) 2–<5 years 5–<10 years >10 years
Concept (policy source)	National United Nations NGO Funder Academic Civil society Other (to add)
Concept (policy type)*	Guidance Strategy Law Plan Protocol Statement Directive Brief Policy Initiative Emergency Development
Context (sector)*	Nutrition Food Child health Child development Maternal health Mental health Reproductive health Neonatal Social care Economic Other (to add)
Population*	Infants under 6 months Small infants under 6 months Sick infants under 6 months Wasted/acutely malnourished infants under 6 months Underweight infants under 6 months Stunted infants under 6 months Growth faltering infants under 6 months Low birth weight infants Other (to add) Mothers All mothers Sick mothers Malnourished mothers Adolescent mothers Other (to add)
Policy definitions	
Policy	Title Reference
Definition status	Yes/no
Population (as applicable)	Infant under 6 months Mother
Definition(s)	Extracted text Text reference Reviewer comment
Strategic interventions	
Intervention	Title Reference
Population (as applicable)	Infant under 6 months Mother
Policy provision(s)	Extracted text Text reference Reviewer comment
Policy alignment, coherence, gaps and opportunities	
Policy	Title Reference
Population (as applicable)	Infant under 6 months Mother
Policy provision(s)	Extracted text Text reference Reviewer comment
Policy development processes (AGREE II components)	Scope and purpose (extracted text) Stakeholder involvement (y/n, details) Procedure for update provided (y/n, details)

*More than one categorisation may apply.

AGREE, Appraisal of Guidelines for Research and Evaluation; NGO, Non-Governmental Organisation.

The MAMI Care Pathway framework^17^ and applicable components of the AGREE II (**A**ppraisal of **G**uidelines for **Re**search and **E**valuation) Tool^29^ will be used to organise and guide appraisal of policy alignment, coherence, opportunities and gaps. This will be undertaken from extracted data by one researcher and assessed in the reviewers’ meeting. Any difference of opinion in the review team at any point will be resolved through discussion within the team. The results of the search and the study inclusion process will be comprehensively described in the scoping review and presented in a flow chart as indicated in the PRISMA-ScR statement.^30^

The review will provide an overview of the type of evidence and data extracted from the relevant policy findings. A basic descriptive analysis of extracted data will be included and mapped visually in figures, tables or diagrams as appropriate. Strategic interventions and policy alignment, coherence, opportunities and gaps will be described in narrative form. Findings will be discussed with in-country stakeholders (at group presentations/interviews) to inform conclusions and recommendations.

### Limitations

No assessment of the quality of the documents included in the scoping review will be carried out as this is not the object of this study. Similarly, the review will not scope research evidence since this is not the objective of the review. A full AGREE II appraisal of identified documents is not feasible. The review is limited to English and Amharic documentation.

### Patient and public involvement

There has been no patient or public involvement in the development of this protocol.

### Ethics and dissemination

#### Ethical considerations

Ethical approval is not needed since the data to be collected consist of published articles and publicly available material. The funders will have no role in conducting the review.

#### Dissemination and uptake

The findings of this review will be shared directly with national and international stakeholders working in child health and nutrition and will be published in an open access journal.

The review will involve coauthorship of national stakeholders, including representation from the Ministry of Health Ethiopia Research Advisory Council^31^ and direct engagement of the research team in national and international networks, fora and guideline processes. An emerging in-country network (the MAMI Country Chapter) will provide primed avenues for dissemination and uptake of evidence-based policies in Ethiopia.

Appraising policy content is an important dimension of policy analysis to support policy uptake of the evidence generated through the MAMI Care Pathway research programme in Ethiopia. The results of this review will be examined by the MAMI RISE Research Team to identify strategic policy targets and key multisectoral initiatives to engage in dissemination of trial findings and with whom to discuss implications for research, policy and practice.

The scoping review will provide evidence on the importance of accounting for national policy context when planning for future scaling of health and nutrition interventions. The rationale and method of scoping the national policy environment will be shared with international stakeholders through the MAMI Global Network, in targeted working groups and presentations, to encourage uptake of consistent and comparable approaches in other contexts.

## Supplementary Material

Reviewer comments

Author's manuscript
