# Supplementary material for: Mapping the range of policies relevant to care of small and nutritionally at-risk infants under 6 months and their mothers in Ethiopia: a scoping review protocol
Source: BMJ Open. 2023 Sep 19;13(9):e069359. doi: 10.1136/bmjopen-2022-069359 (PMC10510927; doi:10.1136/bmjopen-2022-069359)
Supplement: Supplementary data [file bmjopen-2022-069359supp001.pdf]

Supplemental File 1: Search Strategy for OVID Medline

Search Terms	Steps
newborn* or new-born or neonat* or prematur* or infant* or infancy or baby or babies or p?ediatric*	1
exp Infant/	2
1 OR 2 (ALL INFANTS)	3
low-birth-weight or LBW or prematur* or small-for-gestational-age or SGA or small-for-age or SFA	4
exp Infant, Low Birth Weight/ OR exp "Infant, Small for Gestational Age"/ OR exp Infant, Very Low Birth Weight/ OR exp Infant, Premature/	5
4 OR 5 (ALL SMALL)	6
malnourished or malnutrition or severe malnutrition or severely malnourished or severe acute malnutrition or SAM or moderate* malnutrition or moderately malnourished or moderate acute malnutrition or MAM or acute malnutrition or acutely malnourished or AM or severe wasting or severely wasted or moderate wasting or moderately wasted or wasting or wasted or thin* or stunting or stunted or growth-failure or growth-falter* or poor growth or under-weight or failure-to-thrive or FTT or failure-to-grow or growth delay or delayed growth or nutrition*deficien* or micronutrient* deficien* or nutritionally-at-risk or nutrition disorder* or protein-energy-malnutrition or PEM or development* delay or delayed development or mid-upper-arm-circumference or MUAC or weight-for-length or WFL	7
((((exp Child Nutrition Disorders/ OR exp Infant Nutrition Disorders/ OR exp Malnutrition/ OR exp Wasting Syndrome/) OR Nutritional Support/) OR (exp Malnutrition/ OR exp Severe Acute Malnutrition/ OR exp Starvation/ OR exp Wasting Syndrome/)) OR Protein-Energy Malnutrition/) OR (exp Severe Acute Malnutrition/ OR exp Kwashiorkor/)	8
7 OR 8 (ALL NUTRITIONALLY AT RISK)	9
(3 AND 6) OR (3 AND 9) (ALL SMALL INFANTS OR NUTRITIONALLY AT-RISK INFANTS)	10
(mother* or matern*) ADJ2 (health or nutrition or mental-health or reproductive-health or food or social-assistance or social-welfare or nutrition or malnutrition)	11
(((exp Adolescent Mothers/ OR exp Maternal Health/) OR exp Kangaroo-Mother Care Method/) OR exp Maternal Welfare/) OR exp Postnatal Care/	12
11 OR 12 (ALL MOTHERS)	13
10 OR 13 (ALL SMALL INFANTS OR NUTRITIONALLY AT-RISK INFANTS OR MOTHERS)	14
policy or policies or guid* or strateg* or law or legal or plan or protocol* or statement* or directi* or brief or initiative*	15
((((exp Family Planning Policy/ OR exp Health Policy/ OR exp Health Care Reform/ OR exp Nutrition Policy/) OR (exp Policy Making/ OR exp Advisory Committees/)) OR exp Organizational Policy/)) OR (exp Guideline/ OR exp Practice Guideline/)	16
15 OR 16 (ALL POLICIES)	17
14 and 17 (ALL SMALL INFANTS OR NUTRITIONALLY AT-RISK INFANTS OR MOTHERS AND POLICIES)	18
Ethiopia*	19
18 and 19 (ALL SMALL INFANTS OR NUTRITIONALLY AT-RISK INFANTS OR MOTHERS AND POLICIES IN ETHIOPIA)	20
Screen for identified documents specific to infants under six months of age	
