# Supplementary material for: Mapping the range of policies relevant to care of small and nutritionally at-risk infants under 6 months and their mothers in Ethiopia: a scoping review protocol
Source: BMJ Open. 2023 Sep 19;13(9):e069359. doi: 10.1136/bmjopen-2022-069359 (PMC10510927; doi:10.1136/bmjopen-2022-069359)
Supplement: Author's manuscript [file bmjopen-2022-069359.draft_revisions.pdf]

BMJ Open is committed to open peer review. As part of this commitment we make the peer review history of every article we publish publicly available.

When an article is published we post the peer reviewers' comments and the authors' responses online. We also post the versions of the paper that were used during peer review. These are the versions that the peer review comments apply to.

The versions of the paper that follow are the versions that were submitted during the peer review process. They are not the versions of record or the final published versions. They should not be cited or distributed as the published version of this manuscript.

BMJ Open is an open access journal and the full, final, typeset and author-corrected version of record of the manuscript is available on our site with no access controls, subscription charges or pay-per-view fees (http://bmjopen.bmj.com).

If you have any questions on BMJ Open's open peer review process please email info.bmjopen@bmj.com

BMJ Open

Mapping the range of policies relevant to care of small and nutritionally at-risk infants under 6 months and their mothers in Ethiopia: A scoping review protocol

Journal:	*BMJ Open*
Manuscript ID	bmjopen-2022-069359
Article Type:	Protocol
Date Submitted by the Author:	20-Oct-2022
Complete List of Authors:	McGrath, Marie; Emergency Nutrition Network; London School of Hygiene and Tropical Medicine Faculty of Epidemiology and Population Health,<br>Woldie, Mirkuzie; Ethiopia Ministry of Health, Maternal and Child Health and Nutrition Directorate<br>Berhane, Melkamu ; Jimma University, Department of Pediatrics<br>Abera, Mubarek; Jimma University, Faculty of Medical Sciences<br>Hailu, Endashaw; GOAL Ethiopia<br>Rana, Ritu; Indian Institute of Public Health Gandhinagar, Department of Public Health Programmes<br>Lanyero, Betty; World Health Organization Country Office for Ethiopia, Emergency Preparedness and Response Unit<br>Grijalva-Eternod, Carlos ; London School of Hygiene and Tropical Medicine Faculty of Epidemiology and Population Health<br>Abdissa, Alemseged; Armauer Hansen Research Institute<br>Girma, Tsinuel; Jimma University, Department of Pediatrics and Child Health<br>Kerac, Marko; London School of Hygiene and Tropical Medicine Faculty of Epidemiology and Population Health<br>Smythe, Tracey; London School of Hygiene and Tropical Medicine Faculty of Epidemiology and Population Health, International Centre for Evidence in Disability, Department of Population Health,
Keywords:	Nutrition < TROPICAL MEDICINE, Health policy < HEALTH SERVICES ADMINISTRATION & MANAGEMENT, International health services < HEALTH SERVICES ADMINISTRATION & MANAGEMENT, Public health < INFECTIOUS DISEASES

SCHOLARONE™
Manuscripts

Mapping the range of policies relevant to care of small and nutritionally at-risk infants under 6 months and their mothers in Ethiopia: A scoping review protocol

Marie McGrath[1], Mirkuzie Woldie[2], Melkamu Berhane[3], Mubarek Abera[4], Endashaw Hailu[5], Ritu Rana[6], Betty Lanyero[7], Carlos Grijalva-Eternod[8], Alemseged Abdissa[9], Tsinuel Girma[10], Marko Kerac[11], Tracey Smythe[12]

[1]Emergency Nutrition Network, Oxford, UK and Department of Population Health, Faculty of Epidemiology and Population Health, London School of Hygiene and Tropical Medicine, Keppel Street, London WC1E 7HT, UK https://orcid.org/0000-0002-0660-1873

[2]Ministry of Health, Maternal and Child Health and Nutrition Directorate, Addis Ababa, Ethiopia https://orcid.org/0000-0002-1777-0816

[3]Department of Paediatric and Child Health, Jimma University, Jimma P.O. Box 378, Ethiopia https://orcid.org/0000-0003-1517-6220

[4]Faculty of Medical Sciences, Jimma University, Jimma P.O. Box 378, Ethiopia https://orcid.org/0000-0003-2592-1327

[5]GOAL Ethiopia, EW Harargehe program Area, Yeka Subcity, Woreda 9, Addis Ababa P.O. Box 5504, Ethiopia

[6]Department of Public Health Programmes, Indian Institute of Public Health, Gandhinagar 382042, Gujarat, India https://orcid.org/0000-0001-6071-1750

[7]Emergency Preparedness and Response Unit, World Health Organization Country Office for Ethiopia, Addis Ababa, Ethiopia https://orcid.org/0000-0002-8629-7663

[8]Department of Population Health, Faculty of Epidemiology and Population Health, London School of Hygiene and Tropical Medicine, Keppel Street, London WC1E 7HT, UK https://orcid.org/0000-0002-2461-9954

[9]Deputy Director, Armauer Hansen Research Institute, Addis Ababa P.O. Box 1005, Ethiopia https://orcid.org/0000-0003-3798-5037

[10]Department of Pediatrics and Child Health, Jimma University, Jimma, P.O. Box 378, Ethiopia https://orcid.org/0000-0002-3538-0061

[11]Department of Population Health, Faculty of Epidemiology and Population Health, London School of Hygiene and Tropical Medicine, Keppel Street, London WC1E 7HT, UK https://orcid.org/0000-0002-3745-7317

[12]International Centre for Evidence in Disability, Department of Population Health, Faculty of Epidemiology and Population Health, London School of Hygiene and Tropical Medicine, Keppel Street, London WC1E 7HT, UK and Division of Physiotherapy, Department of Health and Rehabilitation Sciences, Stellenbosch University, Cape Town, South Africa https://orcid.org/0000-0003-3408-7362

Corresponding author: Marie McGrath, Emergency Nutrition Network, Oxford, UK. Marie.McGrath@lshtm.ac.uk, +44 (0)7737 996822

For peer review only

Word count: 3392 (excluding title page, abstract, table, references)

ABSTRACT

Introduction

Significant evidence gaps limit management of small and nutritionally at-risk infants under six months and their mothers who are at higher risk of death, illness, malnutrition, poor growth and development. These infants may be low birth weight, wasted, stunted or underweight. An integrated care model to guide their management (MAMI Care Pathway) is being tested in a randomised controlled trial (RCT) in Ethiopia. Evaluating the extent to which an innovation is consistent with existing policies, regulations, national health plans and priorities is a key step in early planning for future scale.

Methods and analysis

This review will evaluate the extent to which the MAMI Care Pathway is consistent with national policies that relate to the care of small and nutritionally at-risk infants under 6 months and their mothers in Ethiopia. The objectives are to describe the range and characteristics, concepts, strategic interventions, coherence and alignment of existing policies and identify opportunities and gaps. It will be conducted in accordance with the JBI methodology for scoping reviews (PRISMA-ScR). Eligible documents include infant and maternal health, nutrition, child development, food and social welfare related policies publicly available in English and Amharic. The protocol was registered on the OSF Registry on 20th June 2022 (https://osf.io/m4jt6).

Grey literature will be identified through government and agency websites, national and sub-national contacts and Google Scholar, and published policies through electronic database searches (MEDLINE, EMBASE, Global and Health Information). A standardized data extraction tool will be used. Descriptive analysis of data will be undertaken and data mapped visually. Tabulated results will be described in narrative form by emergent themes. National stakeholder discussions will inform conclusions and recommendations.

Ethics and dissemination

Ethics approval is not needed since data comprises only publicly available material. Findings will be used to evidence national and international policy and practice.

Strengths and limitations of this study

- A scoping review is most suited to identifying relevant policies given they are characteristically diverse and most likely sourced from in-country grey literature, including through contacts and networks, rather than electronic databases.
- National stakeholders who are directly engaged in policy development and research in Ethiopia are directly involved in the review from conceptualisation.
- The review is consistent with stated strategic intent of the Ethiopian government for multi-sectoral integration of nutrition.
- Critical appraisal of the identified policies is necessary to practically inform national stakeholders and to evidence uptake activities of planned research in Ethiopia.
- The review is limited to English and Amharic documentation.

Keywords: nutrition, health policy, international health services, public health

For peer review only

INTRODUCTION

Globally, about one in five infants under 6 months is small and/or nutritionally at-risk[1]. An estimated 20.1% (23.8 million) infants under 6 months are underweight (low weight-for-age), 17.6% (24.5 million) are wasted/acutely malnourished (low weight-for-length) and 17.6% (21.5 million) are stunted (low length-for-age) [1]. An estimated 9.76% babies are born low birth weight (LBW) in Sub-Saharan Africa[2]. In Ethiopia, an estimated 7% of children under 5 years are wasted and 36.8% are stunted[3], while an estimated 13.2% are born LBW[4]. These infants are at higher risk of death, illness, malnutrition, poor growth and development[5 6].

Community-based management of wasting in children over 6 months of age is a well-established approach globally, involving outpatient and decentralised care for medially uncomplicated cases[7]. Equivalent services for infants under 6 months are not common in low and middle income countries for many reasons including assumptions that infants under 6 months are protected from malnutrition through exclusive breastfeeding[8]. In reality, exclusive breastfeeding prevalence is suboptimal in almost all countries[9] and may be overestimated[10]. In Ethiopia, prevalence amongst infants under 6 months is estimated at 60.4% with regional variation[11]. There remain longstanding evidence gaps in how best to identify and manage cases in outpatient services[8].

Increased awareness of the significant burden of care in this age group led to updated WHO guidance in 2013 recommending outpatient care for medically uncomplicated wasted infants under 6 months[12]. However, uptake of these recommendations into national guidance has been low and slow. A 2020 review of 63 national protocols for malnutrition treatment found that only six countries recommended outpatient care for infants under 6 months [13]. In Ethiopia, like most countries, current national guidelines for treatment of wasting/acute malnutrition in infants under 6 months continue to recommend inpatient care only, which is less accessible for families, limits service coverage and may not fully cater for their needs[14]. Barriers to national policy uptake include demand for more context specific evidence of what works, for whom and at what cost for health systems, services, communities, families and mothers[15]. Lack of community-based options for care means that most small and nutritionally at-risk infants and their mothers do not receive the prompt care that they need, particularly after the early postnatal period (i.e. post six weeks to six months of age) and a critical window of opportunity for early preventive intervention is missed. Experiencing an episode of wasting in early life leads to an increased risk of further wasting in later life [16] which contributes to the global burden of 47 million wasted children [17].

To help operationalize the WHO 2013 recommendations for community-based care for this age group and to fill a gap in operational guidance, the MAMI Care Pathway Package[18] was developed by the MAMI Global Network, an established global collaboration of nutrition and health practitioners, researchers and experts (www.ennonline.net/ourwork/research/mami). This resource material applies an integrated care model to guide management of small and nutritionally at-risk infants under 6 months and their mothers (MAMI). Support to the mother-infant dyad is central to the approach that seeks integrated care in health and nutrition systems and services. It bridges maternal and child nutrition, health, and social interventions to support continuity of quality care, using and building on existing systems and services wherever possible. For example, it includes basic screening for small and nutritionally at-risk infants under six months and mothers at routine immunisation visits and supports implementation of WHO integrated management of childhood illness (IMCI) guidelines in accordance with local protocols.

Global ambitions for integrated services across sectors are shared by the Ethiopian government. The Ethiopian National Food and Nutrition Strategy, for example, highlights multi-sectoral coordination

For peer review only - http://bmjopen.bmj.com/site/about/guidelines.xhtml

and integration is critical but in practice, has been slow and ineffective in bringing about changes to long lasting nutrition and public health problems, in part due to inadequate mainstreaming of nutrition into relevant sectoral policies, strategies, programmes and operational plans[19]. There may be many reasons for this. Sectoral policy development processes and rollout plans are often siloed in different departments/ministries/institutions/agencies missing opportunities to align and collaborate. The concept of small and nutritionally at-risk infants under 6 months and their mothers may also be defined, categorised and understood in different ways across policy makers that may impede cross-sectoral collaboration. Limited alignment of policy provision has consequences at many levels. As well as limiting delivery of strategic ambition, lack of policy coherence also influences the operational context in which health workers work and may impact if, and the degree to which, they have licence and autonomy to integrate and connect different services and specialities on the ground, even when they see the opportunities to do so.

To help fill the critical evidence gap in community based care, the MAMI Care Pathway is being tested in Ethiopia in a north-south research partnership that includes a pragmatic RCT, formative research and process evaluation (www.ennonline.net/ourwork/research/mamiriseethiopia). Findings will be used to evidence national and international policy and practice with the ultimate aim of achieving scalable sustainable care for small and nutritionally at-risk infants under 6 months and their mothers. Understanding, accounting for and responding to the national policy context is critical to ensure that the evidence generated meet the needs of national decision-makers[20 21] and to support the research uptake process. Interventions that correspond to national health-sector goals are likely to gain the political and administrative support necessary for larger scale implementation if the project results demonstrate success[22]. Evaluating the extent to which the innovation is consistent with existing policies, regulations, national health plans and priorities is a key step in early planning for future scale[22].

A scoping review will be undertaken to describe and appraise policies in Ethiopia that address the multiple dimensions of care including nutrition, health, child development, food and social welfare through the lens of managing small and nutritionally at-risk infants under 6 months and their mothers.

METHODS AND ANALYSIS

Design

A scoping review methodology has been selected to allow the collation of a diverse range of relevant information which has not previously been merged in this way[23]. The proposed scoping review will be conducted in accordance with the JBI (formerly Joanna Briggs Institute) methodology for scoping reviews[24]. We have used the JBI Population, Concept and Context (PCC) strategy to define the title, scoping review objective, scoping review question, and inclusion criteria. This review will focus primarily on policy content that will contribute to broader policy analysis that considers policy context, process and actors [25] to support translation of evidence to policy in the context of Ethiopia[26].

Aim and objectives

The aim of this scoping review is to evaluate the extent to which the MAMI Care Pathway is consistent with national policies that relate to the care of small and nutritionally at-risk infants under 6 months and their mothers in Ethiopia. The objectives of the review are to:

For peer review only

- describe the range and characteristics of existing policy documents relevant to the care of small and/or nutritionally at-risk infants under 6 months and their mothers
- describe how small and/or nutritionally at-risk infants under six months and their mothers are conceptualised/defined in policies
- identify strategic interventions targeted at the care of small and nutritionally at-risk infants under six months and/or mothers
- assess alignment, coherence, opportunities and gaps within and across identified policies.

All relevant infant and maternal health, nutrition, child development, food and social welfare related policies and policy documents will be included. To be considered as a policy or policy-related document, we will consider the description provided by Haynes et al. (P. 149)[23], see Box 1.

Box 1: Policy Definitions
Policy: A formal statement or action plan developed by a government agency or statutory body in response to an identified problem. This includes state-wide or national legislation, policies, programs, directives, protocols, guidelines, and service models.

Policy document: A review, report, discussion paper, draft or final policy, formal directive, program plan, strategic plan, ministerial brief, budget bid, service agreement, implementation plan, guideline, or protocol with a focus on health service or program design, delivery, evaluation, or resourcing

Review questions

What is the range and characteristics of policies that relate to the care of small and/or nutritionally at-risk infants under 6 months and their mothers?

1. What is the range and characteristics of policies in Ethiopia that relate to the care of small and/or nutritionally at-risk infants under six months and their mothers?
2. How are small and/or nutritionally at-risk infants under 6 months and their mothers conceptualised, described or defined in Ethiopian policies?
3. What strategic interventions targeted at the care of small and/or nutritionally at-risk infants under six months and mothers are outlined in the identified policies?
4. What alignment, coherence, opportunities and gaps are there in identified policies for the care of small and/or nutritionally at-risk infants under 6 months and their mothers in Ethiopia?

Population (Participants)

This scoping review will focus on individuals (mothers, infants under 6 months, including neonates) across subpopulations (ethnicity, communities) and society in Ethiopia.

Our interest is in those infants under 6 months who are small (e.g. LBW due to prematurity or small-for-gestational age), malnourished or at-risk of malnutrition, rather than the general population of all infants. There is not currently a standard definition of this sub-group but we include those with: LBW; growth faltering; low weight-for-length; low weight-for-age; low length-for-age; low mid upper arm circumference (MUAC); and those whose mothers have health/mental health issues which may affect the infant's care or feeding. These criteria reflect a working definition applied in the MAMI Care Pathway Package (www.ennonlinehttps://www.ennonline.net/ourwork/research/mami). We will also be open to other definitions of infants under 6 months who may be at-risk of the above – the review will help us understand different definitions and links between them better.

For peer review only

Concept

This scoping review of policies aims to identify the main documents that have a bearing on the nature of practice on the care of small and/or nutritionally at-risk infants under 6 months and their mothers in Ethiopia. This review aims to capture all current infant and maternal nutrition, health, child development, food and social welfare -related policy evidence in Ethiopia. We wish to identify policy provisions for both direct and broader/indirect support for the care of mother-infant dyads who are identified as small and/or nutritionally at-risk. Policies that address treatment and/or prevention and that address maternal well-being, economic security and social enterprise, such as social protection and food assistance, will be included in the review. The policy characterisation will conceptually map definitions used, i.e. describe concepts and terminology used to define the population being examined. Policies will be appraised to identify alignment, coherence, opportunities and gaps. Opportunities investigated will include information on policy development processes and the degree to which details (scope and purpose, stakeholder involvement and procedures for update) is available will be reported.

Context

All settings and contexts will be considered eligible that apply to the care of small and/or nutritionally at-risk infants under 6 months and their mothers in Ethiopia. These include but will not be limited to inpatient care, outpatient care, primary health care, communities, and households/families.

Inclusion criteria

Health, nutrition, food, child development, social welfare and other relevant policies in Ethiopia that make provision for care of small and/or nutritionally at-risk infants under 6 months and/or their mothers will be included. National policy documents and World Health Organization (WHO), United Nations (UN), Non-Governmental Organisation (NGO), and funder policies will also be included. Documents published in English and Amharic will be included. No time limit will be set.

Exclusion criteria

Documents focusing on countries others than Ethiopia will be excluded from the review. Documents focusing on the general population of infants u6m who are not small or nutritionally at-risk will also be excluded.

Search Strategy

The development of the search strategy is taking place in collaboration with a librarian. A pilot test of source selectors will be conducted whereby a random sample of abstracts will be screened using the eligibility criteria by the selection team. Discrepancies will be discussed, and clarifications/modifications made to eligibility criteria/definitions accordingly. A preliminary search of Cochrane library, MEDLINE (Ovid), PROSPERO and Open Science Framework (OSF) Registry for systematic and scoping reviews of policies in Ethiopia was conducted on 26th May 2022 and no reviews were found on this review topic. The protocol was registered on the OSF Registry on 20th June 2022 (https://osf.io/m4jt6).

The search strategy will be carried out using the search terms related to each of the concepts of interest. The following search terms and their variants will be used, as split across six concepts: small, nutritionally at-risk, infants under six months, mothers, policies and Ethiopia. The search strategy will combine keywords to include small OR nutritionally at-risk AND infant under 6 months

For peer review only

OR mothers AND policies in Ethiopia. A series of searches will be performed towards a final search to identify policies that relate to small and/or nutritionally at-risk infants under six months and their mothers in Ethiopia.

The scoping review will involve identification of publicly available grey literature and a comprehensive search of three electronic databases for published policies. We anticipate that grey literature will be the primary source of content. Grey literature/database searches will be conducted by one researcher. Reference lists of the included documents will be hand searched to identify further eligible documents.

The scoping review reporting methods outlined by the Preferred Reporting for Items for Systematic reviews and Meta-Analysis Protocols (PRISMA-P) using the scoping review extension (PRISMA- ScR) will be employed. The PRISMA-ScR checklist will be included in the review results paper. Patients and the general public were not involved in the preparation of this protocol. The study will take place between July and November 2022.

Grey literature search

Search terms will be applied as appropriate and additional search terms used and iteratively developed to capture policies that do not specify the concepts of interest and reported on. Google scholar will be used to identify the grey literature and relevant policy documents, reviews or executive summaries. Websites and contacts of the Ethiopian government, MAMI Care Pathway Research Team, WHO, UNICEF, WFP, in-country agencies and funders will be used to acquire relevant information and documents. Policies sourced by the MAMI Care Pathway Research Team during the formative phase of the research programme, including those identified in stakeholder interviews as part of a feasibility study of the MAMI Care Pathway, will be included/updated with the latest version as appropriate.

Electronic database search

Publications related to policy provision for maternal and infant under 6 months care across sectors and services in Ethiopia will be sought using online search engines: MEDLINE (Ovid), EMBASE, Global and Health Information. Search terms will be applied and adapted as appropriate to the syntax of each database. Key research terms will also comprise those obtained via subject headings of searched databases (e.g. Medical Subject Headings (MeSH)). The search strategies for each database will be similar in structure with similar search terms and synonyms. Additional keywords and sources identified during the process will be noted and reported.

Data extraction

All citations of literature to be included in the review will be uploaded into EndNote for deduplication. Results will be exported to Rayan for screening by two independent reviewers. Following an initial pilot each reviewer will independently and systematically screen the title and abstracts of all documents using the inclusion criteria to do so. Documents for review will be limited to the first 50 texts. The full text of documents for possible inclusion in the review will be assessed by the screening team at a reviewers' meeting. Reasons for exclusion of sources of evidence at full text will be logged and then stated in the review itself.

A standardized data extraction tool will be used against the selected policies (see draft charting table in Table 1 below). Extracted data will be organized applying predetermined fields and the themes arising. Basic coding of data to categories, such as sectors, sub-population and by policy type and source will be conducted. Concepts/definitions within a field and policy development information will be extracted. One researcher will carry out data extraction, and the data will be assessed by a

For peer review only

second researcher. The data extraction tool will be changed and adapted during the process of gathering information from the respective evidence source, and all modifications made will be explained fully in the final review.

The MAMI Care Pathway framework[18] and applicable components of the AGREE II (**A**ppraisal of **G**uidelines for **Re**search and **E**valuation) tool[27] will be used to organise and examine details of the policy documents and guide appraisal of their alignment, coherence, opportunities and gaps. This will be undertaken from extracted data by one researcher and assessed in the reviewers meeting. Any difference of opinion in the review team at any point will be resolved through discussion within the team. The results of the search and the study inclusion process will be comprehensively described in the scoping review and presented in a flow chart as indicated in the PRISMA-ScR statement[28].

The review will provide an overview of type of evidence and data extracted from the relevant policy findings. A basic descriptive analysis of extracted data will be included and mapped visually in figures or tables as appropriate. This information will also be presented using diagrams and tables for easy accessibility. Strategic interventions and policy alignment, coherence, opportunities and gaps will be described in narrative form by relevant themes that emerge. Findings will be discussed with in-country stakeholders (group presentation/interviews) to inform conclusions and recommendations.

Table 1: Draft charting table for data extraction

**Parameters**	**Results (n, %)**
**1. Policy characteristics**	
No. of policies identified	Total number
Publication date	<2 years (to current date)<br>2-<5 years<br>5-<10 years<br>>10 years
Concept (Policy source)	National<br>UN<br>NGO<br>Funder<br>Academic<br>Civil society<br>Other (to add)
Concept (Policy type)*	Guidance<br>Strategy<br>Law<br>Plan<br>Protocol<br>Statement<br>Directive<br>Brief<br>Policy<br>Initiative<br>Emergency<br>Development
Context (Sector)*	Nutrition<br>Food

For peer review only - http://bmjopen.bmj.com/site/about/guidelines.xhtml

	Child Health<br>Child Development<br>Maternal Health<br>Mental Health<br>Reproductive Health<br>Neonatal<br>Social care<br>Economic<br>Other (to add)
Population*	Infants under 6 months<br>- Small infants under 6 months<br>- Sick infants under 6 months<br>- Wasted/acutely malnourished infants under 6 months<br>- Underweight infants under 6 months<br>- Stunted infants under 6 months<br>- Growth faltering infants under 6 months<br>- Other (to add)<br>Mothers<br>- All mothers<br>- Sick mothers<br>- Malnourished mothers<br>- Adolescent mothers<br>- Other (to add)
**2. Policy definitions**	
Policy	Title<br>Reference
Definition status	Yes/No
Population (as applicable)	Infant under 6 months<br>Mother
Definition(s)	Extracted text<br>Text reference<br>Reviewer comment
**3. Strategic interventions**	
Intervention	Title<br>Reference
Population (as applicable)	Infant under 6 months<br>Mother
Policy provision(s)	Extracted text<br>Text reference<br>Reviewer comment
**4. Policy alignment, coherence & opportunities**	
Policy	Title<br>Reference
Population (as applicable)	Infant under 6 months<br>Mother
Policy provision(s)	Extracted text<br>Text reference<br>Reviewer comment

For peer review only

Policy development processes (GRADE components)	Scope and purpose (extracted text)<br>Stakeholder involvement (y/n, details)<br>Procedure for update provided (y/n, details)

*More than one categorisation may apply

Limitations

No assessment of the quality of the documents included in the scoping review will be carried out as this is not the object of this study. The review will not scope research evidence since this is not the objective of the review. A full GRADE appraisal of identified documents is not feasible.

Patient and public involvement

There has been no patient or public involvement in the development of this protocol.

ETHICS and DISSEMINATION

Ethical considerations

Ethics approval is not needed since data to be collected consists of published articles and publicly available material. The funders will have no role in conducting the review.

Contribution to knowledge

Mapping the range and characteristics of national policies through the lens of small and nutritionally at-risk infants under 6 months and their mothers in Ethiopia will identify barriers to address, opportunities to leverage and gaps to fill to help mainstream integrated continuity of care across multi-sectoral national policy and service provisions. In doing so, this works seeks to support delivery on and development of strategic priorities of the Ethiopian government, including implementation of the Ethiopian National Food and Nutrition Strategy. It will also inform early planning to help national decision-makers appraise future scale potential[22].

Appraising policy content is an important dimension of policy analysis to support application of the evidence generated through the MAMI Care Pathway research programme in Ethiopia. The process of this review will inform practical actions for the research partnership including identifying strategic policy targets and key multi-sectoral initiatives to engage with.

Co-authorship of national stakeholders in the scoping review including representation from the Ministry of Health Ethiopia Research Advisory Council[29], direct engagement of the research team in national and international networks/fora/guideline processes, and an emerging in-country network (MAMI Country Chapter) will provide primed avenues for policy appraisal, knowledge sharing and help bridge evidence to policy processes in Ethiopia.

The method of scoping the national policy environment will provide practical insight on the 'how' for researchers and service providers in similar contexts and provide evidence on the importance of accounting for national policy context from the outset when planning for future optimal scale of health and nutrition interventions.

Conclusion

The proposed scoping review will ultimately contribute to achieving scalable, sustainable care for small and nutritionally at-risk infants under 6 months and their mothers in Ethiopia and globally.

For peer review only

Acknowledgements

The MAMI research team would like to acknowledge Katie Perrins, LSHTM librarian for her review of the search methodology and Natasha Lelijveld and Philip James, ENN for their review of the search strategy. The team also gratefully acknowledge the funding support of Eleanor Crook Foundation.

Conflicts of interest

No conflict of interest has been declared by the authors.

Authors' contributions

MM, TS, MK and MW conceptualised the scoping review. MM drafted the methodology. AA and TG reviewed the concept, scope, methodology and search strategy. MB, EH, CG, RR and BL reviewed the scope, methodology and search strategy.

Funding statement

This research was funded by the Eleanor Crook Foundation (433PH) and Irish Aid (HQPCR/2022/ENN).

REFERENCES

1. Kerac M, James PT, McGrath MG, et al. Infant malnutrition in low- and middle-income countries: assessment and prevalence of small and nutritionally at-risk infants aged under 6 months in 54 Demographic & Health Survey datasets. *medRxiv* 2021:2021.12.23.21268306. doi: 10.1101/2021.12.23.21268306
2. Tessema ZT, Tamirat KS, Teshale AB, et al. Prevalence of low birth weight and its associated factor at birth in Sub-Saharan Africa: A generalized linear mixed model. *PLOS ONE* 2021;16(3):e0248417. doi: 10.1371/journal.pone.0248417
3. ICF EPHIEEa. Ethiopia Mini Demographic and Health Survey 2019: Final Report. Rockville, Maryland, USA: EPHI and ICF. 2021
4. Central Statistical Agency - CSA/Ethiopia, ICF. Ethiopia Demographic and Health Survey 2016. Addis Ababa, Ethiopia: CSA and ICF, 2017.
5. Mertens A, Benjamin-Chung J, Colford JM, et al. Causes and consequences of child growth failure in low- and middle-income countries. *medRxiv* 2020:2020.06.09.20127100. doi: 10.1101/2020.06.09.20127100
6. Mertens A, Benjamin-Chung J, Colford JM, et al. Child wasting and concurrent stunting in low- and middle-income countries. *medRxiv* 2021:2020.06.09.20126979. doi: 10.1101/2020.06.09.20126979
7. WHO, WFP, UNSCN, et al. Community-based management of severe acute malnutrition. A Joint Statement by the World Health Organization, the World Food Programme, the United Nations System Standing Committee on Nutrition and the United Nations Children's Fund 2007
8. ENN, UCL, ACF. MAMI Project. Technical Review: Current evidence, policies, practices & programme outcomes. Jan 2010. 2010;0:0.
9. Zong Xn, Wu H, Zhao M, et al. Global prevalence of WHO infant feeding practices in 57 LMICs in 2010–2018 and time trends since 2000 for 44 LMICs. *eClinicalMedicine* 2021;37 doi: 10.1016/j.eclinm.2021.100971

For peer review only

10. Mulol H, Coutsoudis A. Limitations of maternal recall for measuring exclusive breastfeeding rates in South African mothers. *Int Breastfeed J* 2018;13:19. doi: 10.1186/s13006-018-0159-8 [published Online First: 2018/06/01]
11. Wake GE, Mittiku YM. Prevalence of exclusive breastfeeding practice and its association with maternal employment in Ethiopia: a systematic review and meta-analysis. *International Breastfeeding Journal* 2021;16(1):86.
12. WHO. WHO Guidelines Approved by the Guidelines Review Committee. Guideline: Updates on the Management of Severe Acute Malnutrition in Infants and Children. Geneva: World Health Organization 2013.
13. Lelijveld N, McGrath M, Mertens A, et al. Growth failure in infants younger than 6 months: a scoping review. Submitted to WHO scoping consultation on growth failure in infants under six months: ENN, 2020.
14. Government of Ethiopia FMoH. National Guideline for the Management of Acute Malnutrition. Addis Ababa: FMOH.
15. Read BS, McGrath M. Community management of uncomplicated malnourished infants under six months old: barriers to national policy change. 2018; (57). https://www.ennonline.net/fex/57/malnourishedinfantschange (accessed 08/04/2018 00:00:00).
16. Mertens A, Benjamin-Chung J, Colford Jr JM, et al. Child wasting and concurrent stunting in low- and middle-income countries. *medRxiv* 2020 doi: doi: https://doi.org/10.1101/2020.06.09.20126979
17. Global Nutrition Report Stakeholder G. 2020 Global Nutrition Report: Action on equity to end malnutrition. Bristol, UK, 2020.
18. MAMI Global Network, ENN, LSHTM. MAMI Care Pathway Package. v3. . *https://wwwennonlinenet/mamicarepathway* 2021
19. Ethiopia. FDRo. National Food and Nutrition Strategy. May 2021
20. Lancaster K, Rhodes T. What prevents health policy being 'evidence-based'? New ways to think about evidence, policy and interventions in health. *Br Med Bull* 2020;135(1):38-49. doi: 10.1093/bmb/ldaa026 [published Online First: 2020/09/09]
21. Erismann S, Pesantes MA, Beran D, et al. How to bring research evidence into policy? Synthesizing strategies of five research projects in low-and middle-income countries. *Health Res Policy Syst* 2021;19(1):29. doi: 10.1186/s12961-020-00646-1 [published Online First: 2021/03/08]
22. WHO, ExpandNet. Beginning with the end in mind. Planning pilot projects and other programmatic research for successful scaling up. 2011
23. Haynes A, Turner T, Redman S, et al. Developing definitions for a knowledge exchange intervention in health policy and program agencies: Reflections on process and value. *International Journal of Social Research Methodology* 2015;18:145-59. doi: 10.1080/13645579.2014.921457
24. Peters MDJ, Marnie C, Tricco AC, et al. Updated methodological guidance for the conduct of scoping reviews. *JBI Evidence Implementation* 2021;19(1):3-10. doi: 10.1097/xeb.0000000000000277
25. Walt G, Gilson L. Reforming the health sector in developing countries: the central role of policy analysis. *Health Policy Plan* 1994;9(4):353-70. doi: 10.1093/heapol/9.4.353 [published Online First: 1994/11/04]
26. Walt G, Shiffman J, Schneider H, et al. 'Doing' health policy analysis: methodological and conceptual reflections and challenges. *Health Policy Plan* 2008;23(5):308-17. doi: 10.1093/heapol/czn024 [published Online First: 2008/08/15]
27. Brouwers MC, Kho ME, Browman GP, et al. AGREE II: advancing guideline development, reporting and evaluation in health care. *Cmaj* 2010;182(18):E839-42. doi: 10.1503/cmaj.090449 [published Online First: 2010/07/07]

For peer review only

28. Tricco AC, Lillie E, Zarin W, et al. PRISMA Extension for Scoping Reviews (PRISMA-ScR): Checklist and Explanation. *Ann Intern Med* 2018;169(7):467-73. doi: 10.7326/m18-0850 [published Online First: 2018/09/05]
29. Woldie M, Yakob B, Berman P, et al. 62 The role of the research advisory council (RAC) in bridging the evidence-to-policy gap in maternal and child health in ethiopia. *BMJ Evidence-Based Medicine* 2019;24(Suppl 1):A36-A36. doi: 10.1136/bmjebm-2019-EBMLive.70

For peer review only

BMJ Open

Mapping the range of policies relevant to care of small and nutritionally at-risk infants under six months and their mothers in Ethiopia: A scoping review protocol

Journal:	*BMJ Open*
Manuscript ID	bmjopen-2022-069359.R1
Article Type:	Protocol
Date Submitted by the Author:	26-Apr-2023
Complete List of Authors:	McGrath, Marie; Emergency Nutrition Network; London School of Hygiene and Tropical Medicine Faculty of Epidemiology and Population Health,<br>Woldie, Mirkuzie; Ethiopia Ministry of Health, Maternal and Child Health and Nutrition Directorate<br>Berhane, Melkamu ; Jimma University, Department of Pediatrics<br>Abera, Mubarek; Jimma University, Faculty of Medical Sciences<br>Hailu, Endashaw; GOAL Ethiopia<br>Rana, Ritu; Indian Institute of Public Health Gandhinagar, Department of Public Health Programmes<br>Lanyero, Betty; World Health Organization Country Office for Ethiopia, Emergency Preparedness and Response Unit<br>Grijalva-Eternod, Carlos ; London School of Hygiene & Tropical Medicine Faculty of Epidemiology and Population Health; University College London, Institute for Global Health<br>Abdissa, Alemseged; Armauer Hansen Research Institute<br>Girma, Tsinuel; Jimma University, Department of Pediatrics and Child Health<br>Kerac, Marko; London School of Hygiene and Tropical Medicine Faculty of Epidemiology and Population Health<br>Smythe, Tracey; London School of Hygiene and Tropical Medicine Faculty of Epidemiology and Population Health, International Centre for Evidence in Disability, Department of Population Health,
**Primary Subject Heading**:	Global health
Secondary Subject Heading:	Health policy, Public health, Nutrition and metabolism, Evidence based practice, Health services research
Keywords:	Health policy < HEALTH SERVICES ADMINISTRATION & MANAGEMENT, International health services < HEALTH SERVICES ADMINISTRATION & MANAGEMENT, Public health < INFECTIOUS DISEASES, Nutrition < TROPICAL MEDICINE

Mapping the range of policies relevant to care of small and nutritionally at-risk infants under six months and their mothers in Ethiopia: A scoping review protocol

Marie McGrath[1], Mirkuzie Woldie[2], Melkamu Berhane[3], Mubarek Abera[4], Endashaw Hailu[5], Ritu Rana[6], Betty Lanyero[7], Carlos Grijalva-Eternod[8], Alemseged Abdissa[9], Tsinuel Girma[10], Marko Kerac[11], and Tracey Smythe[12]

[1] Emergency Nutrition Network, Oxford, UK and Department of Population Health, Faculty of Epidemiology and Population Health, London School of Hygiene and Tropical Medicine, Keppel Street, London, WC1E 7HT, UK https://orcid.org/0000-0002-0660-1873

[2] Ministry of Health, Maternal and Child Health and Nutrition Directorate, Addis Ababa, Ethiopia https://orcid.org/0000-0002-1777-0816

[3] Department of Paediatric and Child Health, Jimma University, Jimma, PO Box 378, Ethiopia https://orcid.org/0000-0003-1517-6220

[4] Faculty of Medical Sciences, Jimma University, Jimma, PO Box 378, Ethiopia https://orcid.org/0000-0003-2592-1327

[5] GOAL Ethiopia, EW Harargehe Program Area, Yeka Subcity, Woreda 9, Addis Ababa, PO Box 5504, Ethiopia

[6] Department of Public Health Programmes, Indian Institute of Public Health, Gandhinagar 382042, Gujarat, India https://orcid.org/0000-0001-6071-1750

[7] Emergency Preparedness and Response Unit, World Health Organization Country Office for Ethiopia, Addis Ababa, Ethiopia https://orcid.org/0000-0002-8629-7663

[8] Department of Population Health, Faculty of Epidemiology and Population Health, London School of Hygiene and Tropical Medicine, Keppel Street, London, WC1E 7HT, UK and Institute for Global Health, University College London, 30 Guilford Street, London, WC1N 1EH, UK https://orcid.org/0000-0002-2461-9954

[9] Deputy Director, Armauer Hansen Research Institute, Addis Ababa, PO Box 1005, Ethiopia https://orcid.org/0000-0003-3798-5037

[10] Department of Pediatrics and Child Health, Jimma University, Jimma, PO Box 378, Ethiopia https://orcid.org/0000-0002-3538-0061

[11] Department of Population Health, Faculty of Epidemiology and Population Health, London School of Hygiene and Tropical Medicine, Keppel Street, London, WC1E 7HT, UK https://orcid.org/0000-0002-3745-7317

[12] International Centre for Evidence in Disability, Department of Population Health, Faculty of Epidemiology and Population Health, London School of Hygiene and Tropical Medicine, Keppel Street, London, WC1E 7HT, UK and Division of Physiotherapy, Department of Health and Rehabilitation Sciences, Stellenbosch University, Cape Town, South Africa https://orcid.org/0000-0003-3408-7362

Corresponding author: Marie McGrath, Emergency Nutrition Network, Oxford, UK. Marie.McGrath@lshtm.ac.uk, +44 (0)7737 996822

For peer review only

Word count: 3523 (excluding title page, abstract, table, references)

ABSTRACT (300)

Introduction

Evidence gaps limit management of small and/or nutritionally at-risk infants under six months and their mothers, who are at higher risk of death, illness, malnutrition, and poor growth and development. These infants may be low birth weight, wasted, stunted and/or underweight. An integrated care model to guide their management (MAMI Care Pathway) is being tested in a randomised controlled trial in Ethiopia. Evaluating the extent to which an innovation is consistent with national policies and priorities will aid evidence uptake and plan for scale.

Methods and analysis

This review will evaluate the extent to which the MAMI Care Pathway is consistent with national policies that relate to the care of at-risk infants under six months and their mothers in Ethiopia. The objectives are to describe the range and characteristics, concepts, strategic interventions, coherence and alignment of existing policies and identify opportunities and gaps. It will be conducted in accordance with the JBI methodology for scoping reviews (PRISMA-ScR). Eligible documents include infant and maternal health, nutrition, child development, food and social welfare-related policies publicly available in English and Amharic. The protocol was registered on the Open Science Framework (OSF) Registry on 20 June 2022 (https://osf.io/m4jt6).

Grey literature will be identified through government and agency websites, national and sub-national contacts and Google Scholar, and published policies through electronic database searches (MEDLINE, EMBASE and Global and Health Information). The searches will take place between May and December 2023. A standardised data-extraction tool will be used. Descriptive analysis of data will be undertaken. Data will be mapped visually and tabulated. Results will be described in narrative form. National stakeholder discussions will inform conclusions and recommendations.

Ethics and dissemination

Ethical approval is not required as data consists solely of publicly available material. Findings will be used to evidence national and international policy and practice.

Strengths and limitations of this study

- A scoping review is most suited to identifying relevant policies given they are characteristically diverse and most likely sourced from in-country grey literature, including through contacts and networks, rather than electronic databases.
- National stakeholders who are directly engaged in policy development and research in Ethiopia are directly involved in the review from conceptualisation onwards.
- The review is consistent with the stated strategic intent of the Ethiopian government for multi-sectoral integration of nutrition.
- Critical appraisal of the identified policies is necessary to practically inform national stakeholders and to evidence uptake activities related to planned research in Ethiopia.
- The review is limited to English and Amharic documentation.

Keywords: nutrition, health policy, international health services, public health

For peer review only

INTRODUCTION

Globally, about one in five infants under six months is small and/or nutritionally at-risk (at-risk)[1]. An estimated 20.1% (23.8 million) infants under six months are underweight (low weight-for-age), 17.6% (24.5 million) are wasted/acutely malnourished (low weight-for-length) and 17.6% (21.5 million) are stunted (low length-for-age)[1]. An estimated 9.76% of babies are born low birth weight (LBW) in Sub-Saharan Africa[2]. In Ethiopia, an estimated 7% of children under five years are wasted and 36.8% are stunted[3], while an estimated 13.2% are born LBW[4]. These infants are at higher risk of death, illness, malnutrition and poor growth and development[5 6]. Furthermore, experiencing an episode of wasting in early life leads to an increased risk of further wasting in later life[6] which contributes to the global burden of 47 million wasted children [7].

Community-based management of wasting in children over six months of age is a well-established approach globally, involving outpatient and decentralised care for medically uncomplicated cases[8]. Equivalent services for infants under six months are not common in low- and middle-income countries for many reasons, including assumptions that infants under six months are protected from malnutrition through exclusive breastfeeding[9]. In reality, exclusive breastfeeding prevalence is suboptimal in almost all countries[10] and may be overestimated[11]. In Ethiopia, prevalence among infants under six months is estimated at 60.4%, with regional variations[12].

Increased awareness of the significant burden of care in this age group led to updated World Health Organization (WHO) guidelines in 2013 recommending outpatient care for uncomplicated severely wasted/acutely malnourished infants under six months[13]. However, uptake of WHO recommendations into national guidance has been low and slow. A 2020 review of 63 national protocols for malnutrition treatment found that only six countries recommended outpatient care for infants under six months [14]. In most countries, including Ethiopia, inpatient treatment remains the only option, which is less accessible for families, limits service coverage and may not fully cater for their needs[15]. Barriers to national policy uptake of WHO guidelines include demand for more context-specific evidence of what works, for whom and at what cost for health systems, services, communities, families and mothers[16]. This means that most at-risk infants and their mothers do not receive the prompt care they need, particularly after the early postnatal period (i.e. post six weeks to six months of age), missing a critical window for early treatment and preventive action.

Longstanding gaps in implementation guidance on how best to identify and manage at-risk infants under six months in outpatient and community services have hampered WHO policy translation into practice[9]. To help address this, the MAMI Care Pathway Package[17] was developed through expert consultation by the MAMI Global Network, an established global collaboration of nutrition and health practitioners, researchers and experts (www.ennonline.net/ourwork/research/mami). This resource material applies a care pathway model to guide practical management of at-risk infants under six months and their mothers. Support to the mother–infant pair is central to the MAMI approach. It bridges maternal and child nutrition, health and social interventions to support continuity of quality, respectful care, integrated into and building on existing health and nutrition systems and services wherever possible. For example, it includes basic screening for at-risk infants under six months and mothers at routine immunisation visits and supports implementation of WHO Integrated Management of Childhood Illness guidelines in accordance with local protocols. The MAMI Care Pathway Package has been implemented in multiple contexts by non-governmental organisations (NGOs) through small-scale programmes and operational research.[18-20] There have been two updates (most recently in 2021) through expert consultation and informed by implementation experiences and the latest evidence.

Global ambitions for integrated services across sectors are shared by the Ethiopian government. The Ethiopian National Food and Nutrition Strategy, for example, highlights multi-sectoral coordination

For peer review only - http://bmjopen.bmj.com/site/about/guidelines.xhtml

and integration as critical, but notes that progress has been slow and ineffective in bringing about changes to nutrition and public health problems. The Strategy suggests this is partly due to inadequate mainstreaming of nutrition into relevant sectoral policies, strategies, programmes and operational plans[21]. Sectoral policy development processes and rollout plans are often siloed in different departments, ministries and institutions, thus missing opportunities to align and collaborate. The concept of at-risk infants under six months and their mothers may be defined and understood in different ways across diverse policymakers, which may itself impede cross-sectoral collaboration. Poor alignment of policy limits achievement of strategic ambition and misses opportunities to leverage collection action. Lack of policy coherence also influences the operational context in which health workers work; siloed services may restrict frontline workers' autonomy and capacity to innovate and connect across services during their day-to-day activities.

To help fill the critical evidence gap in outpatient care, the MAMI Care Pathway is being tested in Ethiopia in a north–south research partnership that includes a pragmatic randomised controlled trial, formative research and a process evaluation (MAMI RISE) (www.ennonline.net/ourwork/research/mamiriseethiopia). Findings will be used to evidence national and international policy and practice, with the ultimate aim of achieving scalable sustainable care for at-risk infants under six months and their mothers. Understanding, accounting for and responding to the national policy context is critical to ensure that the evidence generated meets the needs of national decision-makers[22 23] and to support the research uptake process. Interventions that correspond to national health sector goals are likely to gain the political and administrative support necessary for larger-scale implementation if the project results demonstrate success[24]. Evaluating the extent to which the innovation is consistent with existing policies, regulations, national health plans and priorities is a key step in early planning for future scale[24]. To this end, a scoping review will be undertaken to describe and appraise policies in Ethiopia that address the multiple dimensions of care, including nutrition, health, child development, food and social welfare, through the lens of managing at-risk infants under six months and their mothers.

METHODS AND ANALYSIS

Design

A scoping review methodology has been selected to allow the collation of a diverse range of relevant information not previously merged in this way[25]. The proposed scoping review will be conducted in accordance with the JBI methodology for scoping reviews[26]. We have used the JBI Population, Concept and Context strategy to define the title, scoping review objective, scoping review question and inclusion criteria. This review will focus primarily on policy content that will contribute to broader policy analysis that considers policy context, process and actors [27] to support translation of evidence to policy in the context of Ethiopia[28].

Aim and objectives

The aim of this scoping review is to evaluate the extent to which the MAMI Care Pathway is consistent with national policies that relate to the care of at-risk infants under six months and their mothers in Ethiopia. The objectives of the review are to:

- describe the range and characteristics of existing policy documents relevant to the care of small and/or nutritionally at-risk infants under six months and their mothers;
- describe how small and/or nutritionally at-risk infants under six months and their mothers are conceptualised/defined in policies;
- identify strategic interventions targeted at the care of small and/or nutritionally at-risk infants under six months and/or mothers; and
- assess alignment, coherence, opportunities and gaps within and across the identified policies.

For peer review only - http://bmjopen.bmj.com/site/about/guidelines.xhtml

Mapping the range and characteristics of national policies through the lens of at-risk infants under six months and their mothers in Ethiopia will help identify barriers to address, opportunities to leverage and gaps to fill to help mainstream integrated continuity of care across multi-sectoral national policy and service provisions. This review will inform research dissemination actions and aid evidence uptake. Furthermore, it will support the delivery and development of the strategic priorities of the Ethiopian government, including implementation of the Ethiopian National Food and Nutrition Strategy. It will also inform early planning by national decision-makers and help their appraisal of potential for scaling sustainable integrated care of at-risk infants under six months[24] in the future.

Review questions

1. What is the range and characteristics of policies in Ethiopia that relate to the care of small and/or nutritionally at-risk infants under six months and their mothers?
2. How are small and/or nutritionally at-risk infants under six months and their mothers conceptualised, described or defined in Ethiopian policies?
3. What strategic interventions targeted at the care of small and/or nutritionally at-risk infants under six months and mothers are outlined in the identified policies?
4. What alignment, coherence, opportunities and gaps are there in identified policies for the care of small and/or nutritionally at-risk infants under six months and their mothers in Ethiopia?

All relevant infant and maternal health, nutrition, child development, food and social welfare-related policies and policy documents will be included. To be considered as a policy or policy-related document, we will consider the description provided by Haynes et al. (2015, p. 149)[25] (Box 1). The searches will take place between May and December 2023.

Box 1: Policy definitions

Policy: A formal statement or action plan developed by a government agency or statutory body in response to an identified problem. This includes state-wide or national legislation, policies, programmes, directives, protocols, guidelines and service models.

Policy document: A review, report, discussion paper, draft or final policy, formal directive, programme plan, strategic plan, ministerial brief, budget bid, service agreement, implementation plan, guideline, or protocol with a focus on health service or programme design, delivery, evaluation, or resourcing.

Population (participants)

This scoping review will focus on individuals (mothers, infants under six months, including neonates) across subpopulations (ethnicities, communities) and society in Ethiopia.

Our interest is in those infants under six months who are born small (e.g. LBW, premature, small-for-gestational age) and/or are identified as malnourished or growing poorly or at-risk of malnutrition or poor growth, rather than the general population of all infants. There is not currently a standard definition of this sub-group but we include those with: LBW; prematurity; small-for-gestational age; growth faltering; low weight-for-length; low weight-for-age; low length-for-age; low mid upper arm circumference; and those whose mothers have physical/mental health issues that may affect the infant's care or feeding. These criteria reflect a working definition applied in the MAMI Care Pathway

For peer review only

Package[17]. We will also be open to other definitions of at-risk infants under six months – the review will help us understand different definitions and links between them better.

Concept

This scoping review of policies aims to identify the main documents that have a bearing on the nature of practice regarding the care of at-risk infants under six months and their mothers in Ethiopia. This review aims to capture all current infant and maternal nutrition, health, child development, food and social welfare-related policy evidence in Ethiopia. We wish to identify policy provisions for both direct and broader/indirect support for the care of at-risk mother–infant pairs. Policies that address treatment and/or prevention and that address maternal well-being, economic security and social enterprise, such as social protection and food assistance, will be included in the review. The policy characterisation will conceptually map definitions used, i.e. describe the concepts and terminology used to define the population being examined. Policies will be appraised to identify alignment, coherence, opportunities and gaps. Opportunities investigated will include information on policy development processes, while the degree to which details (scope and purpose, stakeholder involvement and procedures for update) is available will be reported.

Context

All settings and contexts will be considered eligible that apply to the care of at-risk infants under six months and their mothers in Ethiopia. These include but will not be limited to inpatient care, outpatient care, primary health care, communities and households/families.

Inclusion criteria

Health, nutrition, food, child development, social welfare and other relevant policies in Ethiopia that make provision for or that are applicable to the care of at-risk infants under six months and/or their mothers will be included. National policy documents and WHO, United Nations, NGO and funder policies will also be included. Documents published in English and Amharic will be included. No time limit will be set.

Exclusion criteria

Documents focusing on countries others than Ethiopia will be excluded from the review. Documents focusing on the general population of infants under six months who are not at-risk and that are not applicable to at-risk infant/mothers' care will also be excluded.

Search strategy

The development of the search strategy is taking place in collaboration with a librarian. A pilot test of source selectors will be conducted whereby a random sample of abstracts will be screened by the selection team using the eligibility criteria. Discrepancies will be discussed and clarifications/modifications made to eligibility criteria/definitions accordingly. A preliminary search of Cochrane library, MEDLINE (Ovid), PROSPERO and OSF Registry for systematic and scoping reviews of policies in Ethiopia was conducted on 26 May 2022 and no reviews were found on this review topic. The protocol was registered on the OSF Registry on 20 June 2022 (https://osf.io/m4jt6).

The search strategy will be carried out using the search terms related to each of the concepts of interest. The following search terms and their variants will be used, as split across six concepts: small, nutritionally at-risk, infants under six months, mothers, policies and Ethiopia. The search strategy will combine keywords to include small OR nutritionally at-risk AND infant under six months OR mothers AND policies in Ethiopia. A series of searches will be performed towards a final search to identify policies that relate to small and/or nutritionally at-risk infants under six months and their mothers in Ethiopia.

For peer review only

The scoping review will involve the identification of publicly available grey literature and a comprehensive search of three electronic databases for published policies. We anticipate that grey literature will be the primary source of content.

Findings will be reported according to the Preferred Reporting for Items for Systematic reviews and Meta-Analysis Protocols (PRISMA-P) scoping review extension (PRISMA-ScR). The PRISMA-ScR checklist will be included in the review results paper. Patients and the general public were not involved in the preparation of this protocol.

Grey literature search

Search terms will be applied as appropriate and additional search terms used and iteratively developed to capture policies that do not specify the concepts of interest and reported on. Google Scholar will be used to identify the grey literature and relevant policy documents, reviews or executive summaries. Websites and contacts of the Ethiopian government, MAMI RISE Research Team, WHO, UNICEF, World Food Programme, in-country agencies and funders will be used to acquire relevant information and documents. Policies sourced by the MAMI RISE Research Team during the formative phase of the research programme, including those identified in stakeholder interviews as part of a feasibility study of the MAMI Care Pathway, will be included/updated with the latest version as appropriate. Grey literature/database searches will be conducted by one researcher and sources and findings cross-checked by a second national researcher. The search process will be mapped and reported. The reference lists of all included literature will be additionally hand-searched for any further relevant literature.

Electronic database search

Publications related to policy provision for maternal and infant under six months care across sectors and services in Ethiopia will be sought using online search engines: MEDLINE (Ovid), EMBASE and Global and Health Information. Search terms will be applied and adapted as appropriate to the syntax of each database. Key research terms will also comprise those obtained via the subject headings of searched databases (e.g. Medical Subject Headings (MeSH)). The search strategies for each database will be similar in structure, with similar search terms and synonyms. Additional keywords and sources identified during the process will be noted and reported.

Data extraction

All citations of literature to be included in the review will be uploaded into EndNote for deduplication. Results will be exported to Rayan for screening by two independent reviewers. Following an initial pilot, each reviewer will independently and systematically screen the title and abstracts of all documents using the inclusion criteria to do so. Documents for review will be limited to the first 50 texts. The full text of documents for possible inclusion in the review will be assessed by the screening team at a reviewers' meeting. Reasons for exclusion of sources of evidence at full text review will be logged and then stated in the review itself.

A standardised data-extraction tool will be used against the selected policies (see the draft charting table in Table 1 below). Extracted data will be organised applying predetermined fields and the themes arising. Basic coding of data to categories, such as sectors, sub-population and by policy type and source, will be conducted. Concepts/definitions within a field and policy development information will be extracted. One researcher will carry out data extraction and the data will be assessed by a second researcher. The data-extraction tool will be changed and adapted during the process of gathering information from the respective evidence source, and all modifications made will be explained fully in the final review.

For peer review only

The MAMI Care Pathway framework[17] and applicable components of the AGREE II (**A**ppraisal of **G**uidelines for **Re**search and **E**valuation) tool[29] will be used to organise and guide appraisal of policy alignment, coherence, opportunities and gaps. This will be undertaken from extracted data by one researcher and assessed in the reviewers' meeting. Any difference of opinion in the review team at any point will be resolved through discussion within the team. The results of the search and the study inclusion process will be comprehensively described in the scoping review and presented in a flow chart as indicated in the PRISMA-ScR statement[30].

The review will provide an overview of the type of evidence and data extracted from the relevant policy findings. A basic descriptive analysis of extracted data will be included and mapped visually in figures, tables or diagrams as appropriate. Strategic interventions and policy alignment, coherence, opportunities and gaps will be described in narrative form. Findings will be discussed with in-country stakeholders (at group presentations/interviews) to inform conclusions and recommendations.

Table 1: Draft charting table for data extraction

**Parameters**	**Results (n, %)**
**1. Policy characteristics**	
No. of policies identified	Total number
Publication date	<2 years (to current date)<br>2–<5 years<br>5–<10 years<br>>10 years
Concept (policy source)	National<br>United Nations<br>NGO<br>Funder<br>Academic<br>Civil society<br>Other (to add)
Concept (policy type)*	Guidance<br>Strategy<br>Law<br>Plan<br>Protocol<br>Statement<br>Directive<br>Brief<br>Policy<br>Initiative<br>Emergency<br>Development
Context (sector)*	Nutrition<br>Food<br>Child health<br>Child development<br>Maternal health<br>Mental health<br>Reproductive health<br>Neonatal<br>Social care

For peer review only

	Economic<br>Other (to add)
Population*	Infants under six months<br>- Small infants under six months<br>- Sick infants under six months<br>- Wasted/acutely malnourished infants under six months<br>- Underweight infants under six months<br>- Stunted infants under six months<br>- Growth faltering infants under six months<br>- Other (to add)<br>Mothers<br>- All mothers<br>- Sick mothers<br>- Malnourished mothers<br>- Adolescent mothers<br>- Other (to add)
**2. Policy definitions**	
Policy	Title<br>Reference
Definition status	Yes/No
Population (as applicable)	Infant under six months<br>Mother
Definition(s)	Extracted text<br>Text reference<br>Reviewer comment
**3. Strategic interventions**	
Intervention	Title<br>Reference
Population (as applicable)	Infant under six months<br>Mother
Policy provision(s)	Extracted text<br>Text reference<br>Reviewer comment
**4. Policy alignment, coherence, gaps and opportunities**	
Policy	Title<br>Reference
Population (as applicable)	Infant under six months<br>Mother
Policy provision(s)	Extracted text<br>Text reference<br>Reviewer comment
Policy development processes (AGREE II components)	Scope and purpose (extracted text)<br>Stakeholder involvement (y/n, details)<br>Procedure for update provided (y/n, details)

*More than one categorisation may apply

Limitations

No assessment of the quality of the documents included in the scoping review will be carried out as this is not the object of this study. Similarly, the review will not scope research evidence since this is

For peer review only

not the objective of the review. A full AGREE II appraisal of identified documents is not feasible. The review is limited to English and Amharic documentation.

Patient and public involvement

There has been no patient or public involvement in the development of this protocol.

ETHICS AND DISSEMINATION

Ethical considerations

Ethical approval is not needed since the data to be collected consists of published articles and publicly available material. The funders will have no role in conducting the review.

Dissemination and uptake

The findings of this review will be shared directly with national and international stakeholders working in child health and nutrition and will be published in an open access journal.

The review will involve co-authorship of national stakeholders, including representation from the Ministry of Health Ethiopia Research Advisory Council[31] and direct engagement of the research team in national and international networks, fora and guideline processes. An emerging in-country network (the MAMI Country Chapter) will provide primed avenues for dissemination and uptake of evidence-based policies in Ethiopia.

Appraising policy content is an important dimension of policy analysis to support policy uptake of the evidence generated through the MAMI Care Pathway research programme in Ethiopia. The results of this review will be examined by the MAMI RISE Research Team to identify strategic policy targets and key multi-sectoral initiatives to engage in dissemination of trial findings and with whom to discuss implications for research, policy and practice.

The scoping review will provide evidence on the importance of accounting for national policy context when planning for future scaling of health and nutrition interventions. The rationale and method of scoping the national policy environment will be shared with international stakeholders through the MAMI Global Network, in targeted working groups and presentations, to encourage uptake of consistent and comparable approaches in other contexts.

Acknowledgements

The MAMI research team would like to acknowledge Katie Perrins (LSHTM librarian) for her review of the search methodology and Natasha Lelijveld and Philip James (ENN) for their review of the search strategy. The team also gratefully acknowledge the funding support of Eleanor Crook Foundation and Irish Aid.

Conflicts of interest

No conflict of interest has been declared by the authors.

Authors' contributions

MM, TS, MK and MW conceptualised the scoping review. MM drafted the methodology. AA and TG reviewed the concept, scope, methodology and search strategy. MB, MA, EH, CG, RR and BL reviewed the scope, methodology and search strategy.

Funding statement

For peer review only

This research was funded by the Eleanor Crook Foundation (433PH) and Irish Aid (HQPCR/2022/ENN).

REFERENCES

1. Kerac M, James PT, McGrath MG, et al. Infant malnutrition in low- and middle-income countries: assessment and prevalence of small and nutritionally at-risk infants aged under 6 months in 54 Demographic Health Survey datasets. *medRxiv* 2021:2021.12.23.21268306. doi: 10.1101/2021.12.23.21268306
2. Tessema ZT, Tamirat KS, Teshale AB, et al. Prevalence of low birth weight and its associated factor at birth in Sub-Saharan Africa: A generalized linear mixed model. *PLOS ONE* 2021;16(3):e0248417. doi: 10.1371/journal.pone.0248417
3. Ethiopian Public Health Institute (EPHI) [Ethiopia], ICF. Ethiopia Mini Demographic and Health Survey 2019: Final Report. Rockville, Maryland, USA: EPHI and ICF. 2021
4. Central Statistical Agency - CSA/Ethiopia, ICF. Ethiopia Demographic and Health Survey 2016. Addis Ababa, Ethiopia: CSA and ICF, 2017.
5. Mertens A, Benjamin-Chung J, Colford JM, et al. Causes and consequences of child growth failure in low- and middle-income countries. *medRxiv* 2020:2020.06.09.20127100. doi: 10.1101/2020.06.09.20127100
6. Mertens A, Benjamin-Chung J, Colford JM, et al. Child wasting and concurrent stunting in low- and middle-income countries. *medRxiv* 2021:2020.06.09.20126979. doi: 10.1101/2020.06.09.20126979
7. Global Nutrition Report Stakeholder G. 2020 Global Nutrition Report: Action on equity to end malnutrition. Bristol, UK, 2020.
8. WHO, WFP, UNSCN, et al. Community-based management of severe acute malnutrition. A Joint Statement by the World Health Organization, the World Food Programme, the United Nations System Standing Committee on Nutrition and the United Nations Children's Fund 2007
9. ENN, UCL, ACF. MAMI Project. Technical Review: Current evidence, policies, practices & programme outcomes. Jan 2010. 2010
10. Zong Xn, Wu H, Zhao M, et al. Global prevalence of WHO infant feeding practices in 57 LMICs in 2010-2018 and time trends since 2000 for 44 LMICs. *eClinicalMedicine* 2021;37 doi: 10.1016/j.eclinm.2021.100971
11. Mulol H, Coutsoudis A. Limitations of maternal recall for measuring exclusive breastfeeding rates in South African mothers. *Int Breastfeed J* 2018;13:19. doi: 10.1186/s13006-018-0159-8 [published Online First: 2018/06/01]
12. Wake GE, Mittiku YM. Prevalence of exclusive breastfeeding practice and its association with maternal employment in Ethiopia: a systematic review and meta-analysis. *International Breastfeeding Journal* 2021;16(1):86.
13. WHO. WHO Guidelines Approved by the Guidelines Review Committee. Guideline: Updates on the Management of Severe Acute Malnutrition in Infants and Children. Geneva: World Health Organization 2013.
14. Lelijveld N, McGrath M, Mertens A, et al. Growth failure in infants younger than 6 months: a scoping review. Submitted to WHO scoping consultation on growth failure in infants under six months: ENN, 2020.
15. Government of Ethiopia FMoH. National Guideline for the Management of Acute Malnutrition. Addis Ababa: FMOH. 2019
16. Read BS, McGrath M. Community management of uncomplicated malnourished infants under six months old: barriers to national policy change. 2018; (57). https://www.ennonline.net/fex/57/malnourishedinfantschange (accessed 08/04/2018 00:00:00).

17. MAMI Global Network, ENN, LSHTM. MAMI Care Pathway Package. v3. 2021
18. Munirul Islam M, Arafat Y, Connell N, et al. Severe malnutrition in infants aged <6 months-Outcomes and risk factors in Bangladesh: A prospective cohort study. *Matern Child Nutr* 2019;15(1):e12642. doi: 10.1111/mcn.12642 [published Online First: 2018/07/05]
19. Butler S, Connell N, Barthorp H. C-MAMI tool evaluation: Learnings from Bangladesh and Ethiopia. 2018; (58). www.ennonline.net/fex/58/cmamitoolevaluation.
20. Beck K, Kirk C, Bradford J, et al. The Paediatric Development Clinic: A model to improve medical, nutritional and developmental outcomes for high-risk children aged under-five in rural Rwanda. 2018; (58). www.ennonline.net/fex/58/thepaediatricdevelopmentclinic.
21. Ethiopia. FDRo. National Food and Nutrition Strategy. 2021
22. Lancaster K, Rhodes T. What prevents health policy being 'evidence-based'? New ways to think about evidence, policy and interventions in health. *Br Med Bull* 2020;135(1):38-49. doi: 10.1093/bmb/ldaa026 [published Online First: 2020/09/09]
23. Erismann S, Pesantes MA, Beran D, et al. How to bring research evidence into policy? Synthesizing strategies of five research projects in low-and middle-income countries. *Health Res Policy Syst* 2021;19(1):29. doi: 10.1186/s12961-020-00646-1 [published Online First: 2021/03/08]
24. WHO, ExpandNet. Beginning with the end in mind. Planning pilot projects and other programmatic research for successful scaling up. 2011
25. Haynes A, Turner T, Redman S, et al. Developing definitions for a knowledge exchange intervention in health policy and program agencies: Reflections on process and value. *International Journal of Social Research Methodology* 2015;18:145-59. doi: 10.1080/13645579.2014.921457
26. Peters MDJ, Marnie C, Tricco AC, et al. Updated methodological guidance for the conduct of scoping reviews. *JBI Evidence Implementation* 2021;19(1):3-10. doi: 10.1097/xeb.0000000000000277
27. Walt G, Gilson L. Reforming the health sector in developing countries: the central role of policy analysis. *Health Policy Plan* 1994;9(4):353-70. doi: 10.1093/heapol/9.4.353 [published Online First: 1994/11/04]
28. Walt G, Shiffman J, Schneider H, et al. 'Doing' health policy analysis: methodological and conceptual reflections and challenges. *Health Policy Plan* 2008;23(5):308-17. doi: 10.1093/heapol/czn024 [published Online First: 2008/08/15]
29. Brouwers MC, Kho ME, Browman GP, et al. AGREE II: advancing guideline development, reporting and evaluation in health care. *CMAJ* 2010;182(18):E839-42. doi: 10.1503/cmaj.090449 [published Online First: 2010/07/07]
30. Tricco AC, Lillie E, Zarin W, et al. PRISMA Extension for Scoping Reviews (PRISMA-ScR): Checklist and Explanation. *Ann Intern Med* 2018;169(7):467-73. doi: 10.7326/m18-0850 [published Online First: 2018/09/05]
31. Woldie M, Yakob B, Berman P, et al. The role of the research advisory council (RAC) in bridging the evidence-to-policy gap in maternal and child health in Ethiopia. *BMJ Evidence-Based Medicine* 2019;24(Suppl 1):A36-A36. doi: 10.1136/bmjebm-2019-EBMLive.70

For peer review only

BMJ Open

Mapping the range of policies relevant to care of small and nutritionally at-risk infants under six months and their mothers in Ethiopia: A scoping review protocol

Journal:	*BMJ Open*
Manuscript ID	bmjopen-2022-069359.R2
Article Type:	Protocol
Date Submitted by the Author:	15-May-2023
Complete List of Authors:	McGrath, Marie; Emergency Nutrition Network; London School of Hygiene and Tropical Medicine Faculty of Epidemiology and Population Health,<br>Woldie, Mirkuzie; Ethiopia Ministry of Health, Maternal and Child Health and Nutrition Directorate<br>Berhane, Melkamu ; Jimma University, Department of Pediatrics<br>Abera, Mubarek; Jimma University, Faculty of Medical Sciences<br>Hailu, Endashaw; GOAL Ethiopia<br>Rana, Ritu; Indian Institute of Public Health Gandhinagar, Department of Public Health Programmes<br>Lanyero, Betty; World Health Organization Country Office for Ethiopia, Emergency Preparedness and Response Unit<br>Grijalva-Eternod, Carlos ; London School of Hygiene & Tropical Medicine Faculty of Epidemiology and Population Health; University College London, Institute for Global Health<br>Abdissa, Alemseged; Armauer Hansen Research Institute<br>Girma, Tsinuel; Jimma University, Department of Pediatrics and Child Health<br>Kerac, Marko; London School of Hygiene and Tropical Medicine Faculty of Epidemiology and Population Health<br>Smythe, Tracey; London School of Hygiene and Tropical Medicine Faculty of Epidemiology and Population Health, International Centre for Evidence in Disability, Department of Population Health,
**Primary Subject Heading**:	Global health
Secondary Subject Heading:	Health policy, Public health, Nutrition and metabolism, Evidence based practice, Health services research
Keywords:	Health policy < HEALTH SERVICES ADMINISTRATION & MANAGEMENT, International health services < HEALTH SERVICES ADMINISTRATION & MANAGEMENT, Public health < INFECTIOUS DISEASES, Nutrition < TROPICAL MEDICINE

Mapping the range of policies relevant to care of small and nutritionally at-risk infants under six months and their mothers in Ethiopia: A scoping review protocol.

Marie McGrath[1], Mirkuzie Woldie[2], Melkamu Berhane[3], Mubarek Abera[4], Endashaw Hailu[5], Ritu Rana[6], Betty Lanyero[7], Carlos Grijalva-Eternod[8], Alemseged Abdissa[9], Tsinuel Girma[10], Marko Kerac[11], and Tracey Smythe[12]

[1] Emergency Nutrition Network, Oxford, UK and Department of Population Health, Faculty of Epidemiology and Population Health, London School of Hygiene and Tropical Medicine, Keppel Street, London, WC1E 7HT, UK https://orcid.org/0000-0002-0660-1873

[2] Ministry of Health, Maternal and Child Health and Nutrition Directorate, Addis Ababa, Ethiopia https://orcid.org/0000-0002-1777-0816

[3] Department of Paediatric and Child Health, Jimma University, Jimma, PO Box 378, Ethiopia https://orcid.org/0000-0003-1517-6220

[4] Faculty of Medical Sciences, Jimma University, Jimma, PO Box 378, Ethiopia https://orcid.org/0000-0003-2592-1327

[5] GOAL Ethiopia, EW Harargehe Program Area, Yeka Subcity, Woreda 9, Addis Ababa, PO Box 5504, Ethiopia

[6] Department of Public Health Programmes, Indian Institute of Public Health, Gandhinagar 382042, Gujarat, India https://orcid.org/0000-0001-6071-1750

[7] Emergency Preparedness and Response Unit, World Health Organization Country Office for Ethiopia, Addis Ababa, Ethiopia https://orcid.org/0000-0002-8629-7663

[8] Department of Population Health, Faculty of Epidemiology and Population Health, London School of Hygiene and Tropical Medicine, Keppel Street, London, WC1E 7HT, UK and Institute for Global Health, University College London, 30 Guilford Street, London, WC1N 1EH, UK https://orcid.org/0000-0002-2461-9954

[9] Deputy Director, Armauer Hansen Research Institute, Addis Ababa, PO Box 1005, Ethiopia https://orcid.org/0000-0003-3798-5037

[10] Department of Pediatrics and Child Health, Jimma University, Jimma, PO Box 378, Ethiopia https://orcid.org/0000-0002-3538-0061

[11] Department of Population Health, Faculty of Epidemiology and Population Health, London School of Hygiene and Tropical Medicine, Keppel Street, London, WC1E 7HT, UK https://orcid.org/0000-0002-3745-7317

[12] International Centre for Evidence in Disability, Department of Population Health, Faculty of Epidemiology and Population Health, London School of Hygiene and Tropical Medicine, Keppel Street, London, WC1E 7HT, UK and Division of Physiotherapy, Department of Health and Rehabilitation Sciences, Stellenbosch University, Cape Town, South Africa https://orcid.org/0000-0003-3408-7362

Corresponding author: Marie McGrath, Emergency Nutrition Network, Oxford, UK. Marie.McGrath@lshtm.ac.uk, +44 (0)7737 996822

Word count: 3566 (excluding title page, abstract, table, references)

For peer review only

ABSTRACT (300)

Introduction

Evidence gaps limit management of small and/or nutritionally at-risk infants under six months and their mothers, who are at higher risk of death, illness, malnutrition, and poor growth and development. These infants may be low birth weight, wasted, stunted and/or underweight. An integrated care model to guide their management (MAMI Care Pathway) is being tested in a randomised controlled trial in Ethiopia. Evaluating the extent to which an innovation is consistent with national policies and priorities will aid evidence uptake and plan for scale.

Methods and analysis

This review will evaluate the extent to which the MAMI Care Pathway is consistent with national policies that relate to the care of at-risk infants under six months and their mothers in Ethiopia. The objectives are to describe the range and characteristics, concepts, strategic interventions, coherence, and alignment of existing policies and identify opportunities and gaps. It will be conducted in accordance with the JBI methodology for scoping reviews (PRISMA-ScR). Eligible documents include infant and maternal health, nutrition, child development, food and social welfare-related policies publicly available in English and Amharic. The protocol was registered on the Open Science Framework (OSF) Registry on 20 June 2022 (https://osf.io/m4jt6).

Grey literature will be identified through government and agency websites, national and sub-national contacts and Google Scholar, and published policies through electronic database searches (MEDLINE, EMBASE and Global and Health Information). The searches will take place between May and December 2023. A standardised data-extraction tool will be used. Descriptive analysis of data will be undertaken. Data will be mapped visually and tabulated. Results will be described in narrative form. National stakeholder discussions will inform conclusions and recommendations.

Ethics and dissemination

Ethical approval is not required as data consists solely of publicly available material. Findings will be used to evidence national and international policy and practice.

Strengths and limitations of this study

- A scoping review is most suited to identifying relevant policies given they are characteristically diverse and most likely sourced from in-country grey literature, including through contacts and networks, rather than electronic databases.
- National stakeholders who are directly engaged in policy development and research in Ethiopia are directly involved in the review from conceptualisation onwards.
- The review is consistent with the stated strategic intent of the Ethiopian government for multi-sectoral integration of nutrition.
- Critical appraisal of the identified policies is necessary to practically inform national stakeholders and to evidence uptake activities related to planned research in Ethiopia.
- The review is limited to English and Amharic documentation.

Keywords: nutrition, health policy, international health services, public health

For peer review only

INTRODUCTION

Globally, about one in five infants under six months is small and/or nutritionally at-risk (at-risk)(1). An estimated 20.1% (23.8 million) infants under six months are underweight (low weight-for-age), 17.6% (24.5 million) are wasted/acutely malnourished (low weight-for-length) and 17.6% (21.5 million) are stunted (low length-for-age)(1). An estimated 9.76% of babies are born low birth weight (LBW) in Sub-Saharan Africa(2). In Ethiopia, an estimated 7% of children under five years are wasted and 36.8% are stunted(3), while an estimated 13.2% are born LBW(4). These infants are at higher risk of death, illness, malnutrition and poor growth and development(5, 6). Furthermore, experiencing an episode of wasting in early life leads to an increased risk of further wasting in later life(6) which contributes to the global burden of 47 million wasted children (7).

Community-based management of wasting in children over six months of age is a well-established approach globally, involving outpatient and decentralised care for medically uncomplicated cases(8). Equivalent services for infants under six months are not common in low- and middle-income countries for many reasons, including assumptions that infants under six months are protected from malnutrition through exclusive breastfeeding(9). In reality, exclusive breastfeeding prevalence is suboptimal in almost all countries(10) and may be overestimated(11). In Ethiopia, prevalence among infants under six months is estimated at 60.4%, with regional variations(12).

Increased awareness of the significant burden of care in this age group led to updated World Health Organization (WHO) guidelines in 2013 recommending outpatient care for uncomplicated severely wasted/acutely malnourished infants under six months(13). However, uptake of WHO recommendations into national guidance has been low and slow. A 2020 review of 63 national protocols for malnutrition treatment found that only six countries recommended outpatient care for infants under six months (14). In most countries, including Ethiopia, inpatient treatment remains the only option, which is less accessible for families, limits service coverage and may not fully cater for their needs(15). Barriers to national policy uptake of WHO guidelines include demand for more context-specific evidence of what works, for whom and at what cost for health systems, services, communities, families and mothers(16). This means that most at-risk infants and their mothers do not receive the prompt care they need, particularly after the early postnatal period (i.e. post six weeks to six months of age), missing a critical window for early treatment and preventive action.

Longstanding gaps in implementation guidance on how best to identify and manage at-risk infants under six months in outpatient and community services have hampered WHO policy translation into practice(9). To help address this, the MAMI Care Pathway Package(17) was developed through expert consultation by the MAMI Global Network, an established global collaboration of nutrition and health practitioners, researchers and experts (www.ennonline.net/ourwork/research/mami). This resource material applies a care pathway model to guide practical management of at-risk infants under six months and their mothers. Support to the mother–infant pair is central to the MAMI approach. It bridges maternal and child nutrition, health and social interventions to support continuity of quality, respectful care, integrated into and building on existing health and nutrition systems and services wherever possible. For example, it includes basic screening for at-risk infants under six months and mothers at routine immunisation visits and supports implementation of WHO Integrated Management of Childhood Illness guidelines in accordance with local protocols. The MAMI Care Pathway Package has been implemented in multiple contexts by non-governmental organisations (NGOs) through small-scale programmes and operational research.(18-20) There have been two updates (most recently in 2021) through expert consultation and informed by implementation experiences and the latest evidence.

Global ambitions for integrated services across sectors are shared by the Ethiopian government. The Ethiopian National Food and Nutrition Strategy, for example, highlights multi-sectoral coordination

For peer review only

and integration as critical, but notes that progress has been slow and ineffective in bringing about changes to nutrition and public health problems. The Strategy suggests this is partly due to inadequate mainstreaming of nutrition into relevant sectoral policies, strategies, programmes and operational plans(21). Sectoral policy development processes and rollout plans are often siloed in different departments, ministries and institutions, thus missing opportunities to align and collaborate. The concept of at-risk infants under six months and their mothers may be defined and understood in different ways across diverse policymakers, which may itself impede cross-sectoral collaboration. Poor alignment of policy limits achievement of strategic ambition and misses opportunities to leverage collection action. Lack of policy coherence also influences the operational context in which health workers work; siloed services may restrict frontline workers' autonomy and capacity to innovate and connect across services during their day-to-day activities.

To help fill the critical evidence gap in outpatient care, the MAMI Care Pathway is being tested in Ethiopia in a north–south research partnership that includes a pragmatic randomised controlled trial, formative research and a process evaluation (MAMI RISE) (www.ennonline.net/ourwork/research/mamiriseethiopia). Findings will be used to evidence national and international policy and practice, with the goal of achieving scalable sustainable care for at-risk infants under six months and their mothers. Understanding, accounting for and responding to the national policy context is critical to ensure that the evidence generated meets the needs of national decision-makers(22, 23) and to support the research uptake process. Interventions that correspond to national health sector goals are likely to gain the political and administrative support necessary for larger-scale implementation if the project results demonstrate success(24). Evaluating the extent to which the innovation is consistent with existing policies, regulations, national health plans and priorities is a key step in early planning for future scale(24). To this end, a scoping review will be undertaken to describe and appraise policies in Ethiopia that address the multiple dimensions of care, including nutrition, health, child development, food, and social welfare, through the lens of managing at-risk infants under six months and their mothers.

METHODS AND ANALYSIS

Design

A scoping review methodology has been selected to allow the collation of a diverse range of relevant information not previously merged in this way(25). The proposed scoping review will be conducted in accordance with the JBI methodology for scoping reviews(26). We have used the JBI Population, Concept and Context strategy to define the title, scoping review objective, scoping review question and inclusion criteria. This review will focus primarily on policy content that will contribute to broader policy analysis that considers policy context, process and actors (27) to support translation of evidence to policy in the context of Ethiopia(28).

Aim and objectives

The aim of this scoping review is to evaluate the extent to which the MAMI Care Pathway is consistent with national policies that relate to the care of at-risk infants under six months and their mothers in Ethiopia. The objectives of the review are to:

- describe the range and characteristics of existing policy documents relevant to the care of small and/or nutritionally at-risk infants under six months and their mothers;
- describe how small and/or nutritionally at-risk infants under six months and their mothers are conceptualised/defined in policies;
- identify strategic interventions targeted at the care of small and/or nutritionally at-risk infants under six months and/or mothers; and
- assess alignment, coherence, opportunities, and gaps within and across the identified policies.

For peer review only

Mapping the range and characteristics of national policies through the lens of at-risk infants under six months and their mothers in Ethiopia will help identify barriers to address, opportunities to leverage and gaps to fill to help mainstream integrated continuity of care across multi-sectoral national policy and service provisions. This review will inform research dissemination actions and aid evidence uptake. Furthermore, it will support the delivery and development of the strategic priorities of the Ethiopian government, including implementation of the Ethiopian National Food and Nutrition Strategy. It will also inform early planning by national decision-makers and help their appraisal of potential for scaling sustainable integrated care of at-risk infants under six months(24) in the future.

Review questions

1. What is the range and characteristics of policies in Ethiopia that relate to the care of small and/or nutritionally at-risk infants under six months and their mothers?
2. How are small and/or nutritionally at-risk infants under six months and their mothers conceptualised, described, or defined in Ethiopian policies?
3. What strategic interventions targeted at the care of small and/or nutritionally at-risk infants under six months and mothers are outlined in the identified policies?
4. What alignment, coherence, opportunities, and gaps are there in identified policies for the care of small and/or nutritionally at-risk infants under six months and their mothers in Ethiopia?

All relevant infant and maternal health, nutrition, child development, food and social welfare-related policies and policy documents will be included. To be considered as a policy or policy-related document, we will consider the description provided by Haynes et al. (2015, p. 149)(25) (Box 1). The searches will take place between May and December 2023.

Box 1: Policy definitions
Policy: A formal statement or action plan developed by a government agency or statutory body in response to an identified problem. This includes state-wide or national legislation, policies, programmes, directives, protocols, guidelines and service models.

Policy document: A review, report, discussion paper, draft or final policy, formal directive, programme plan, strategic plan, ministerial brief, budget bid, service agreement, implementation plan, guideline, or protocol with a focus on health service or programme design, delivery, evaluation, or resourcing.

For peer review only

Population (participants)
This scoping review will focus on individuals (mothers, infants under six months, including neonates) across subpopulations (ethnicities, communities) and society in Ethiopia.

Our interest is in those infants under six months who are born small (e.g., LBW, premature, small-for-gestational age) and/or are identified as malnourished or growing poorly or at-risk of malnutrition or poor growth, rather than the general population of all infants. There is not currently a standard definition of this sub-group, but we include those with: LBW; prematurity; small-for-gestational age; growth faltering; low weight-for-length; low weight-for-age; low length-for-age; low mid upper arm circumference; and those whose mothers have physical/mental health issues that may affect the infant's care or feeding. These criteria reflect a working definition applied in the

MAMI Care Pathway Package(17). We will also be open to other definitions of at-risk infants under six months – the review will help us understand different definitions and links between them better.

Concept

This scoping review of policies aims to identify the main documents that have a bearing on the nature of practice regarding the care of at-risk infants under six months and their mothers in Ethiopia. This review aims to capture all current infant and maternal nutrition, health, child development, food, and social welfare-related policy evidence in Ethiopia. We wish to identify policy provisions for both direct and broader/indirect support for the care of at-risk mother–infant pairs. Policies that address treatment and/or prevention and that address maternal well-being, economic security, and social enterprise, such as social protection and food assistance, will be included in the review. The policy characterisation will conceptually map definitions used, i.e., describe the concepts and terminology used to define the population being examined. Policies will be appraised to identify alignment, coherence, opportunities, and gaps. Opportunities investigated will include information on policy development processes, while the degree to which details (scope and purpose, stakeholder involvement and procedures for update) is available will be reported.

Context

All settings and contexts will be considered eligible that apply to the care of at-risk infants under six months and their mothers in Ethiopia. These include but will not be limited to inpatient care, outpatient care, primary health care, communities and households/families.

Inclusion criteria

Health, nutrition, food, child development, social welfare and other relevant policies in Ethiopia that make provision for or that are applicable to the care of at-risk infants under six months and/or their mothers will be included. National policy documents and WHO, United Nations, NGO and funder policies will also be included. Documents published in English and Amharic will be included. No time limit will be set.

Exclusion criteria

Documents focusing on countries others than Ethiopia will be excluded from the review. Documents focusing on the general population of infants under six months who are not at-risk and that are not applicable to at-risk infant/mothers' care will also be excluded.

Search strategy

The development of the search strategy is taking place in collaboration with a librarian. A pilot test of source selectors will be conducted whereby a random sample of abstracts will be screened by the selection team using the eligibility criteria. Discrepancies will be discussed and clarifications/modifications made to eligibility criteria/definitions accordingly. A preliminary search of Cochrane Library, MEDLINE (Ovid), PROSPERO and OSF Registry for systematic and scoping reviews of policies in Ethiopia was conducted on 26 May 2022 and no reviews were found on this review topic. The protocol was registered on the OSF Registry on 20 June 2022 (https://osf.io/m4jt6).

The search strategy will be carried out using the search terms related to each of the concepts of interest. The following search terms and their variants will be used, as split across six concepts: small, nutritionally at-risk, infants under six months, mothers, policies, and Ethiopia. The search strategy will combine keywords to include small OR nutritionally at-risk AND infant under six months OR mothers AND policies in Ethiopia. A series of searches will be performed towards a final search to identify policies that relate to small and/or nutritionally at-risk infants under six months and their mothers in Ethiopia. See online supplemental file 1 for the initial search terms (MeSH terms and key

For peer review only

words) using MEDLINE (Ovid) platform that will be further adapted to other databases/grey literature sources as described below.

The scoping review will involve the identification of publicly available grey literature and a comprehensive search of three electronic databases for published policies. We anticipate that grey literature will be the primary source of content.

Findings will be reported according to the Preferred Reporting for Items for Systematic reviews and Meta-Analysis Protocols (PRISMA-P) scoping review extension (PRISMA-ScR). The PRISMA-ScR checklist will be included in the review results paper. Patients and the public were not involved in the preparation of this protocol.

Grey literature search

Search terms will be applied as appropriate (see supplemental file 1) and additional search terms iteratively developed and used to capture policies that do not specify the concepts of interest and reported on. Google Scholar will be used to identify the grey literature and relevant policy documents, reviews, or executive summaries. Websites and contacts of the Ethiopian government, MAMI RISE Research Team, WHO, UNICEF, World Food Programme, in-country agencies, and funders will be used to acquire relevant information and documents. Policies sourced by the MAMI RISE Research Team during the formative phase of the research programme, including those identified in stakeholder interviews as part of a feasibility study of the MAMI Care Pathway, will be included/updated with the latest version as appropriate. Grey literature/database searches will be conducted by one researcher and sources and findings cross-checked by a second national researcher. The search process will be mapped and reported. The reference lists of all included literature will be additionally hand-searched for any further relevant literature.

Electronic database search

Publications related to policy provision for maternal and infant under six months care across sectors and services in Ethiopia will be sought using online search engines: MEDLINE (Ovid), EMBASE and Global and Health Information. Search terms will be applied and adapted as appropriate to the syntax of each database. Key research terms will also comprise those obtained via the subject headings of searched databases (e.g., Medical Subject Headings (MeSH)). See supplemental file 1 for MEDLINE (Ovid) search terms. The search strategies for each database will be similar in structure, with similar search terms and synonyms. Additional keywords and sources identified during the process will be noted and reported.

Data extraction

All citations of literature to be included in the review will be uploaded into EndNote for deduplication. Results will be exported to Rayan for screening by two independent reviewers. Following an initial pilot, each reviewer will independently and systematically screen the title and abstracts of all documents using the inclusion criteria to do so. Documents for review will be limited to the first 50 texts. The full text of documents for possible inclusion in the review will be assessed by the screening team at a reviewers' meeting. Reasons for exclusion of sources of evidence at full text review will be logged and then stated in the review itself.

A standardised data-extraction tool will be used against the selected policies (see the draft charting table in Table 1 below). Extracted data will be organised applying predetermined fields and the themes arising. Basic coding of data to categories, such as sectors, sub-population and by policy type and source, will be conducted. Concepts/definitions within a field and policy development information will be extracted. One researcher will carry out data extraction and the data will be assessed by a second researcher. The data-extraction tool will be changed and adapted during the

For peer review only

process of gathering information from the respective evidence source, and all modifications made will be explained fully in the final review.

The MAMI Care Pathway framework(17) and applicable components of the AGREE II (**A**ppraisal of **G**uidelines for **Re**search and **E**valuation) tool(29) will be used to organise and guide appraisal of policy alignment, coherence, opportunities and gaps. This will be undertaken from extracted data by one researcher and assessed in the reviewers' meeting. Any difference of opinion in the review team at any point will be resolved through discussion within the team. The results of the search and the study inclusion process will be comprehensively described in the scoping review and presented in a flow chart as indicated in the PRISMA-ScR statement(30).

The review will provide an overview of the type of evidence and data extracted from the relevant policy findings. A basic descriptive analysis of extracted data will be included and mapped visually in figures, tables or diagrams as appropriate. Strategic interventions and policy alignment, coherence, opportunities, and gaps will be described in narrative form. Findings will be discussed with in-country stakeholders (at group presentations/interviews) to inform conclusions and recommendations.

Table 1: Draft charting table for data extraction

**Parameters**	**Results (n, %)**
**1. Policy characteristics**	
No. of policies identified	Total number
Publication date	<2 years (to current date)<br>2–<5 years<br>5–<10 years<br>>10 years
Concept (policy source)	National<br>United Nations<br>NGO<br>Funder<br>Academic<br>Civil society<br>Other (to add)
Concept (policy type)*	Guidance<br>Strategy<br>Law<br>Plan<br>Protocol<br>Statement<br>Directive<br>Brief<br>Policy<br>Initiative<br>Emergency<br>Development
Context (sector)*	Nutrition<br>Food<br>Child health<br>Child development<br>Maternal health

For peer review only

	Mental health<br>Reproductive health<br>Neonatal<br>Social care<br>Economic<br>Other (to add)
Population*	Infants under six months<br>- Small infants under six months<br>- Sick infants under six months<br>- Wasted/acutely malnourished infants under six months<br>- Underweight infants under six months<br>- Stunted infants under six months<br>- Growth faltering infants under six months<br>- Other (to add)<br>Mothers<br>- All mothers<br>- Sick mothers<br>- Malnourished mothers<br>- Adolescent mothers<br>- Other (to add)
**2. Policy definitions**	
Policy	Title<br>Reference
Definition status	Yes/No
Population (as applicable)	Infant under six months<br>Mother
Definition(s)	Extracted text<br>Text reference<br>Reviewer comment
**3. Strategic interventions**	
Intervention	Title<br>Reference
Population (as applicable)	Infant under six months<br>Mother
Policy provision(s)	Extracted text<br>Text reference<br>Reviewer comment
**4. Policy alignment, coherence, gaps and opportunities**	
Policy	Title<br>Reference
Population (as applicable)	Infant under six months<br>Mother
Policy provision(s)	Extracted text<br>Text reference<br>Reviewer comment
Policy development processes (AGREE II components)	Scope and purpose (extracted text)<br>Stakeholder involvement (y/n, details)<br>Procedure for update provided (y/n, details)

*More than one categorisation may apply

For peer review only

Limitations

No assessment of the quality of the documents included in the scoping review will be carried out as this is not the object of this study. Similarly, the review will not scope research evidence since this is not the objective of the review. A full AGREE II appraisal of identified documents is not feasible. The review is limited to English and Amharic documentation.

Patient and public involvement

There has been no patient or public involvement in the development of this protocol.

ETHICS AND DISSEMINATION

Ethical considerations

Ethical approval is not needed since the data to be collected consists of published articles and publicly available material. The funders will have no role in conducting the review.

Dissemination and uptake

The findings of this review will be shared directly with national and international stakeholders working in child health and nutrition and will be published in an open access journal.

The review will involve co-authorship of national stakeholders, including representation from the Ministry of Health Ethiopia Research Advisory Council(31) and direct engagement of the research team in national and international networks, fora and guideline processes. An emerging in-country network (the MAMI Country Chapter) will provide primed avenues for dissemination and uptake of evidence-based policies in Ethiopia.

Appraising policy content is an important dimension of policy analysis to support policy uptake of the evidence generated through the MAMI Care Pathway research programme in Ethiopia. The results of this review will be examined by the MAMI RISE Research Team to identify strategic policy targets and key multi-sectoral initiatives to engage in dissemination of trial findings and with whom to discuss implications for research, policy, and practice.

The scoping review will provide evidence on the importance of accounting for national policy context when planning for future scaling of health and nutrition interventions. The rationale and method of scoping the national policy environment will be shared with international stakeholders through the MAMI Global Network, in targeted working groups and presentations, to encourage uptake of consistent and comparable approaches in other contexts.

Acknowledgements

The MAMI research team would like to acknowledge Katie Perrins (LSHTM librarian) for her review of the search methodology and Natasha Lelijveld and Philip James (ENN) for their review of the search strategy. The team also gratefully acknowledge the funding support of Eleanor Crook Foundation and Irish Aid.

Conflicts of interest

No conflict of interest has been declared by the authors.

Authors' contributions

MM, TS, MK, and MW conceptualised the scoping review. MM drafted the methodology. AA and TG reviewed the concept, scope, methodology and search strategy. MB, MA, EH, CG-E, RR and BL reviewed the scope, methodology and search strategy.

For peer review only

Funding statement
This research was funded by the Eleanor Crook Foundation (433PH) and Irish Aid (HQPCR/2022/ENN).

REFERENCES

1. Kerac M, James PT, McGrath MG, Brennan E, Opondo C, Frison S. Infant malnutrition in low- and middle-income countries: assessment and prevalence of small and nutritionally at-risk infants aged under 6 months in 54 Demographic Health Survey datasets. medRxiv. 2021:2021.12.23.21268306.
2. Tessema ZT, Tamirat KS, Teshale AB, Tesema GA. Prevalence of low birth weight and its associated factor at birth in Sub-Saharan Africa: A generalized linear mixed model. PLOS ONE. 2021;16(3):e0248417.
3. Ethiopian Public Health Institute (EPHI) [Ethiopia], ICF. Ethiopia Mini Demographic and Health Survey 2019: Final Report. Rockville, Maryland, USA: EPHI and ICF. 2021.
4. Central Statistical Agency - CSA/Ethiopia, ICF. Ethiopia Demographic and Health Survey 2016. Addis Ababa, Ethiopia: CSA and ICF; 2017.
5. Mertens A, Benjamin-Chung J, Colford JM, Coyle J, van der Laan MJ, Hubbard AE, et al. Causes and consequences of child growth failure in low- and middle-income countries. medRxiv. 2020:2020.06.09.20127100.
6. Mertens A, Benjamin-Chung J, Colford JM, Hubbard AE, van der Laan MJ, Coyle J, et al. Child wasting and concurrent stunting in low- and middle-income countries. medRxiv. 2021:2020.06.09.20126979.
7. Global Nutrition Report Stakeholder G. 2020 Global Nutrition Report: Action on equity to end malnutrition. Bristol, UK; 2020. Report No.: 978-1-9164452-7-7.
8. WHO, WFP, UNSCN, UNICEF. Community-based management of severe acute malnutrition. A Joint Statement by the World Health Organization, the World Food Programme, the United Nations System Standing Committee on Nutrition and the United Nations Children's Fund 2007.
9. ENN, UCL, ACF. MAMI Project. Technical Review: Current evidence, policies, practices & programme outcomes. Jan 2010. 2010.
10. Zong Xn, Wu H, Zhao M, Magnussen CG, Xi B. Global prevalence of WHO infant feeding practices in 57 LMICs in 2010-2018 and time trends since 2000 for 44 LMICs. eClinicalMedicine. 2021;37.
11. Mulol H, Coutsoudis A. Limitations of maternal recall for measuring exclusive breastfeeding rates in South African mothers. Int Breastfeed J. 2018;13:19.
12. Wake GE, Mittiku YM. Prevalence of exclusive breastfeeding practice and its association with maternal employment in Ethiopia: a systematic review and meta-analysis. International Breastfeeding Journal. 2021;16(1):86.
13. WHO. WHO Guidelines Approved by the Guidelines Review Committee. Guideline: Updates on the Management of Severe Acute Malnutrition in Infants and Children. Geneva: World Health Organization; 2013.
14. Lelijveld N, McGrath M, Mertens A, Mwangome M, Talbert A, Rana R, et al. Growth failure in infants younger than 6 months: a scoping review. Submitted to WHO scoping consultation on growth failure in infants under six months: ENN; 2020.
15. Government of Ethiopia FMoH. National Guideline for the Management of Acute Malnutrition. Addis Ababa: FMOH. 2019.
16. Read BS, McGrath M. Community management of uncomplicated malnourished infants under six months old: barriers to national policy change2018 08/04/2018 00:00:00 [cited Yemen, Malawi and Vietnam; (57):[27 p.]. Available from: https://www.ennonline.net/fex/57/malnourishedinfantschange.

For peer review only

17. MAMI Global Network, ENN, LSHTM. MAMI Care Pathway Package. v3. 2021.
18. Munirul Islam M, Arafat Y, Connell N, Mothabbir G, McGrath M, Berkley JA, et al. Severe malnutrition in infants aged <6 months-Outcomes and risk factors in Bangladesh: A prospective cohort study. Matern Child Nutr. 2019;15(1):e12642.
19. Butler S, Connell N, Barthorp H. C-MAMI tool evaluation: Learnings from Bangladesh and Ethiopia2018 [cited Bangladesh; Ethiopia; (58):[62 p.]. Available from: www.ennonline.net/fex/58/cmamitoolevaluation.
20. Beck K, Kirk C, Bradford J, Mutaganzwa C, Nahimana E, Bigirumwami O. The Paediatric Development Clinic: A model to improve medical, nutritional and developmental outcomes for high-risk children aged under-five in rural Rwanda2018 [cited Rwanda; (58):[59 p.]. Available from: www.ennonline.net/fex/58/thepaediatricdevelopmentclinic.
21. Ethiopia. FDRo. National Food and Nutrition Strategy. 2021.
22. Lancaster K, Rhodes T. What prevents health policy being 'evidence-based'? New ways to think about evidence, policy and interventions in health. Br Med Bull. 2020;135(1):38-49.
23. Erismann S, Pesantes MA, Beran D, Leuenberger A, Farnham A, Berger Gonzalez de White M, et al. How to bring research evidence into policy? Synthesizing strategies of five research projects in low-and middle-income countries. Health Res Policy Syst. 2021;19(1):29.
24. WHO, ExpandNet. Beginning with the end in mind. Planning pilot projects and other programmatic research for successful scaling up. 2011.
25. Haynes A, Turner T, Redman S, Milat A, Moore G. Developing definitions for a knowledge exchange intervention in health policy and program agencies: Reflections on process and value. International Journal of Social Research Methodology. 2015;18:145-59.
26. Peters MDJ, Marnie C, Tricco AC, Pollock D, Munn Z, Alexander L, et al. Updated methodological guidance for the conduct of scoping reviews. JBI Evidence Implementation. 2021;19(1):3-10.
27. Walt G, Gilson L. Reforming the health sector in developing countries: the central role of policy analysis. Health Policy Plan. 1994;9(4):353-70.
28. Walt G, Shiffman J, Schneider H, Murray SF, Brugha R, Gilson L. 'Doing' health policy analysis: methodological and conceptual reflections and challenges. Health Policy Plan. 2008;23(5):308-17.
29. Brouwers MC, Kho ME, Browman GP, Burgers JS, Cluzeau F, Feder G, et al. AGREE II: advancing guideline development, reporting and evaluation in health care. CMAJ. 2010;182(18):E839-42.
30. Tricco AC, Lillie E, Zarin W, O'Brien KK, Colquhoun H, Levac D, et al. PRISMA Extension for Scoping Reviews (PRISMA-ScR): Checklist and Explanation. Ann Intern Med. 2018;169(7):467-73.
31. Woldie M, Yakob B, Berman P, Herlburt S. The role of the research advisory council (RAC) in bridging the evidence-to-policy gap in maternal and child health in Ethiopia. BMJ Evidence-Based Medicine. 2019;24(Suppl 1):A36-A.

For peer review only

Supplemental File 1: Search Strategy for OVID Medline

Search Terms	Steps
newborn* or new-born or neonat* or prematur* or infant* or infancy or baby or babies or p?ediatric*	1
exp Infant/	2
1 OR 2 (ALL INFANTS)	3
low-birth-weight or LBW or prematur* or small-for-gestational-age or SGA or small-for-age or SFA	4
exp Infant, Low Birth Weight/ OR exp "Infant, Small for Gestational Age"/ OR exp Infant, Very Low Birth Weight/ OR exp Infant, Premature/	5
4 OR 5 (ALL SMALL)	6
malnourished or malnutrition or severe malnutrition or severely malnourished or severe acute malnutrition or SAM or moderate* malnutrition or moderately malnourished or moderate acute malnutrition or MAM or acute malnutrition or acutely malnourished or AM or severe wasting or severely wasted or moderate wasting or moderately wasted or wasting or wasted or thin* or stunting or stunted or growth-failure or growth-falter* or poor growth or under-weight or failure-to-thrive or FTT or failure-to-grow or growth delay or delayed growth or nutrition*deficien* or micronutrient* deficien* or nutritionally-at-risk or nutrition disorder* or protein-energy-malnutrition or PEM or development* delay or delayed development or mid-upper-arm-circumference or MUAC or weight-for-length or WFL	7
((((exp Child Nutrition Disorders/ OR exp Infant Nutrition Disorders/ OR exp Malnutrition/ OR exp Wasting Syndrome/) OR Nutritional Support/) OR (exp Malnutrition/ OR exp Severe Acute Malnutrition/ OR exp Starvation/ OR exp Wasting Syndrome/)) OR Protein-Energy Malnutrition/) OR (exp Severe Acute Malnutrition/ OR exp Kwashiorkor/)	8
7 OR 8 (ALL NUTRITIONALLY AT RISK)	9
(3 AND 6) OR (3 AND 9) (ALL SMALL INFANTS OR NUTRITIONALLY AT-RISK INFANTS)	10
(mother* or matern*) ADJ2 (health or nutrition or mental-health or reproductive-health or food or social-assistance or social-welfare or nutrition or malnutrition)	11
(((exp Adolescent Mothers/ OR exp Maternal Health/) OR exp Kangaroo-Mother Care Method/) OR exp Maternal Welfare/) OR exp Postnatal Care/	12
11 OR 12 (ALL MOTHERS)	13
10 OR 13 (ALL SMALL INFANTS OR NUTRITIONALLY AT-RISK INFANTS OR MOTHERS)	14
policy or policies or guid* or strateg* or law or legal or plan or protocol* or statement* or directi* or brief or initiative*	15
((((exp Family Planning Policy/ OR exp Health Policy/ OR exp Health Care Reform/ OR exp Nutrition Policy/) OR (exp Policy Making/ OR exp Advisory Committees/)) OR exp Organizational Policy/)) OR (exp Guideline/ OR exp Practice Guideline/)	16
15 OR 16 (ALL POLICIES)	17
14 and 17 (ALL SMALL INFANTS OR NUTRITIONALLY AT-RISK INFANTS OR MOTHERS AND POLICIES)	18
Ethiopia*	19
18 and 19 (ALL SMALL INFANTS OR NUTRITIONALLY AT-RISK INFANTS OR MOTHERS AND POLICIES IN ETHIOPIA)	20
Screen for identified documents specific to infants under six months of age	

For peer review only
